# Host–guest complexes of conformationally flexible *C*-hexyl-2-bromoresorcinarene and aromatic *N*-oxides: solid-state, solution and computational studies

**DOI:** 10.3762/bjoc.14.146

**Published:** 2018-07-10

**Authors:** Rakesh Puttreddy, Ngong Kodiah Beyeh, S Maryamdokht Taimoory, Daniel Meister, John F Trant, Kari Rissanen

**Affiliations:** 1 University of Jyvaskyla, Department of Chemistry, P. O. Box 35, 40014 Jyväskylä, Finland; 2 Department of Chemistry, Oakland University, 146 Library Drive, Rochester, Michigan 48309-4479, USA; 3 Department of Chemistry and Biochemistry, University of Windsor, 401 Sunset Avenue, Windsor, N9B 3P4, Canada

**Keywords:** aromatic *N*-oxides, C–H···π Interactions, ditopic receptors, *endo*/*exo* complexation, host–guest chemistry, resorcinarenes

## Abstract

Host–guest complexes of *C*-hexyl-2-bromoresorcinarene (BrC6) with twelve potential aromatic *N*-oxide guests were studied using single crystal X-ray diffraction analysis and ^1^H NMR spectroscopy. In the solid state, of the nine obtained X-ray crystal structures, eight were consistent with the formation of BrC6-*N*-oxide *endo* complexes. The lone exception was from the association between 4-phenylpyridine *N-*oxide and BrC6, in that case the host forms a self-inclusion complex. BrC6, as opposed to more rigid previously studied *C*-ethyl-2-bromoresorcinarene and *C*-propyl-2-bromoresorcinarene, undergoes remarkable cavity conformational changes to host different *N*-oxide guests through C–H···π_(host)_ interactions. In solution phase CD_3_OD/CDCl_3_ (1:1 v/v), all twelve *N*-oxide guests form *endo* complexes according to ^1^H NMR; however, in more polar CD_3_OD/DMSO-*d*_6_ (9:1 v/v), only three *N*-oxides with electron-donating groups form solution-phase *endo* complexes with BrC6. In solid-state studies, 3-methylpyridine *N*-oxide+BrC6 crystallises with both the upper- and lower-rim BrC6 cavities occupied by *N*-oxide guests. Computational DFT-based studies support that lower-rim long hexyl chains provide the additional stability required for this ditopic behaviour. The lower-rim cavity, far from being a neutral hydrophobic environment, is a highly polarizable electrostatically positive surface, aiding in the binding of polar guests such as *N*-oxides.

## Introduction

Resorcinarenes are macrocyclic compounds with a bowl-shaped cavity stabilised by circular intramolecular O···H–O hydrogen bonds (HBs) [1–2]. The combination of their confined cavity and conformational flexibility has driven the interest in these synthetic receptors [3], a subclass of calixarenes [4], for a wide range of applications in fields such as catalysis [5–9], sensors [10–11], coordination chemistry [12–13], biological systems [14] and especially for host–guest (H–G) chemistry [15]. Resorcinarenes can be modified at either the upper rim 2-position, lower rim, or both, to deliver supramolecular structures with the required structure for a given function [16–18]. We have shown that resorcinarenes are particularly suited hosts for both neutral and protonated *N*-heterocyclic compounds [19–20] and alkyl ammonium cations [21–25]. The resulting complexes have been extensively studied in both solid and solution state. The most common defined interactions involve encapsulation in the bowl-shaped upper rim (*endo* complexation) due to size complementarity between host cavity and guest shape, and are generally stabilised through multiple C–H···π interactions [26–28]. The cavity capacity to undergo induced conformational changes in response to the incorporation of various upper-rim substituents, differing lower-rim alkyl chain length, specific guests, and selective solvents, have made resorcinarenes an attractive platform for H–G applications. Through careful rational supramolecular design via self-assembly processes, our lab and others have combined simple 1:1 H–G building blocks into dimers [29–31], hexamers [32] or supramolecular chains (1D), sheets (2D), or lattice (3D) networks [15]. The detailed analysis of the molecular level interactions of these systems also has enabled our research to design constructs with specific individual molecular and electronic properties by tuning the structure of the interacting partners.

**Figure 1:**
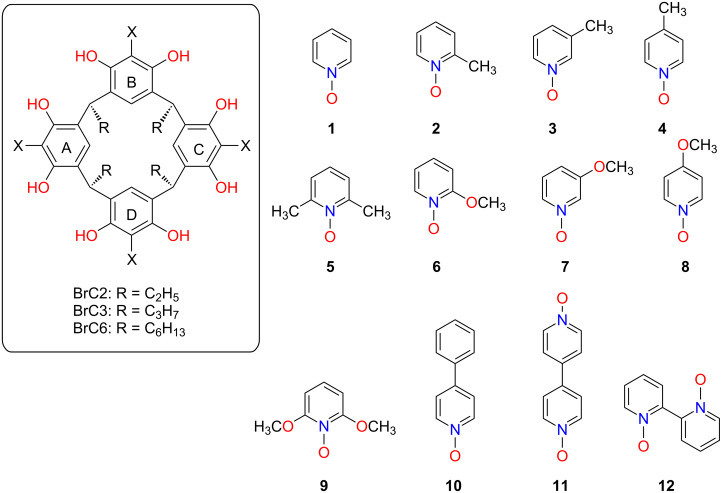
The chemical structures of *C*-ethyl-2-bromoresorcinarene (BrC2), *C*-propyl-2-bromoresorcinarene (BrC3) and *C*-hexyl-2-bromoresorcinarene (BrC6) as hosts and pyridine *N*-oxide (**1**), 2-methylpyridine *N*-oxide (**2**), 3-methylpyridine *N*-oxide (**3**), 4-methylpyridine *N*-oxide (**4**), 2,6-dimethylpyridine *N*-oxide (**5**), 2-methoxypyridine *N*-oxide (**6**), 3-methoxypyridine *N*-oxide (**7**), 4-methoxypyridine *N*-oxide (**8**), 2,6-dimethoxypyridine *N-*oxide (**9**), 4-phenylpyridine *N*-oxide (**10**), 4,4'-bipyridine *N,N*'-dioxide (**11**) and 2,2'-bipyridine *N,N*'-dioxide (**12**) as guests.

Over the past decade, the *N*-oxide family has attracted the attention of the H–G community in molecular recognition processes [33–35]. In order to tune the resorcinarene-PyNO H–G recognition events at the molecular level, a better understanding of the particular interactions is required. The *N*-oxide oxygen atoms potential to act as a HB acceptor for multiple simultaneous N–O···(O–H)_host_ interactions raises the molecular complexity. These are the dominant non-covalent interactions, in both the solid and solution state, compared to *endo* cavity C–H···π_(host)_ interactions that win in the presence of most other guests. Therefore, investigating H–G complexes relying on N–O···(H–O)_host_ HBs is challenging especially in HB competitive solvents such as methanol and dimethyl sulfoxide (DMSO). In reports from our lab, we disclosed that the π-acidity of aromatic protons assist in orienting the *N-*oxide guest by C–H···π interactions, and that the HB accepting N–O group is positioned “up”, extending out beyond the cavity to interact with solvent molecules. Our work, investigating the interactions of PyNO guests with various resorcinarene hosts, has investigated the impact of host cavity flexibility, guest’s steric and electronic demands, and solvent effects, in both solution and the solid state [36–38]. For example, we recently studied *C*-ethyl-2-bromoresorcinarene (BrC2) [39] and *C*-propyl-2-bromoresorcinarene (BrC3) [40] to understand the effect of the electronic nature of the host cavity core and rigidity of the resorcinarene skeleton on the ability to host various PyNO guests. All of these studies have been focused on interactions between the guest and the host upper-rim cavity, either as *endo* guests or as *exo* complexes. However, in these studies, we have occasionally observed interactions between *N-*oxide hosts and the cavity formed by the lower rim alkyl chains. This cavity is well-known to provide additional binding sites for guest molecules [40–41]. Inspired, in the present study, we have investigated the H–G complexes of *C*-hexyl-2-bromoresorcinarene (BrC6) and twelve PyNO guest molecules (Figure 1). The incorporation of long chains in the lower rim creates a hydrophobic secondary lower-rim cavity. This provides the potential for the formation of simultaneous upper- and lower-rim *endo* complexes.

## Results and Discussion

### Solid-state X-ray crystallography

**Figure 2:**
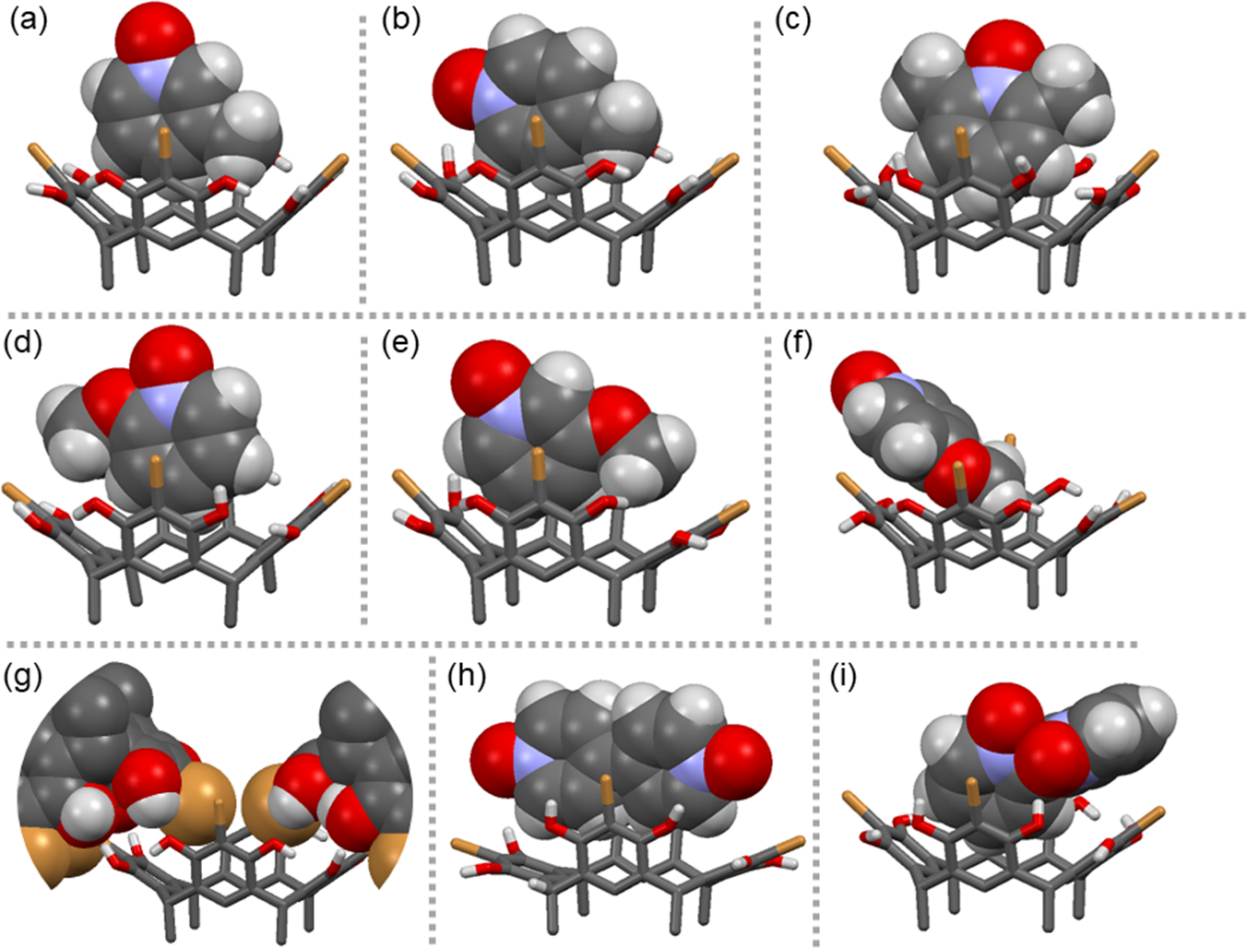
X-ray crystal structures of (a) **3**@BrC6, (b) **4**@BrC6, (c) **5**@BrC6, (d) **6**@BrC6, (e) **7**@BrC6, (f) **8**@BrC6, (g) BrC6 obtained from **10**+BrC6, (h) **11**@BrC6, and (i) **12**@BrC6. The *endo* cavity *N*-oxide guests are shown in CPK models, and the host in capped-stick models. The lower-rim alkyl chains and selected hydrogen atoms were omitted for viewing clarity.

Nine X-ray crystal structures were obtained from BrC6 in combination with twelve PyNO guest molecules. Several attempts to obtain single crystals of BrC6 by itself, **1**+BrC6, **2**+BrC6 and **9**+BrC6 in methanol were unsuccessful. In the following discussions, for example, **1**+BrC6 indicates either from combination of guest **1** and BrC6 or *exo* complex while **1**@BrC6 denotes the *endo* complexation process. However, considering the host flexibility, ‘Δ’ (Table 1), which is the measure of difference between centroid-to-centroid distances of opposite host aromatic rings, guests **1**, **2**, and **9** should easily fit into BrC6 cavity for *endo* complexation processes. The lack of a crystal structure for these systems should consequently not imply that they do not encapsulate. The Δ values for BrC6 in H–G complexes are >1.0 Å (Table 1) and range between 1.08 Å and 2.39 Å, which are relatively high when compared to BrC2 (range, 0.08–1.06 Å) and BrC3 (range, 0.32–1.81 Å) values. In solid-state crystals, the lower-rim hexyl chains which prefer different orientations due to C–C bond flexibility cause BrC6 to crystallise as non-centrosymmetric hosts in all H–G complexes. In our previous PyNO–BrC2 complexes, more than 50% of BrC2 hosts are centrosymmetric [39]. In other words, long lower-rim hexyl chains cause the high Δ values observed for BrC6, which facilitates a remarkably flexible cavity for various guests. For the following discussions, the position of the guest inside the BrC6 cavity is represented as ‘*h*’, defined as the measured distance from the centroid of the lower-rim host carbon atoms to the nearest *endo* guest non-hydrogen atom. In the X-ray structure of **3**@BrC6 (Figure 2a), guest **3**, oriented parallel to the host aromatic rings (*h* = 3.43 Å) is positioned in one corner of the cavity with only the proton *meta*- to the N–O group interacting with a host aromatic ring. This short contact C–H···π_(host)_ interaction is about 2.65–2.85 Å long. In **4**@BrC6 (Figure 2b), once again guest **4** is oriented parallel to the host aromatic rings (*h* = 3.38 Å) and the H–G recognition occurs by C–H···π_(host)_ interaction at two sites through C2 proton (2.49–2.89 Å) and methyl group hydrogen atoms (2.93–3.0 Å). This behaviour is in contrast with H–G complex **4**@BrC2, where the BrC2 rigid cavity only allows the methyl group of **4** to insert inside the cavity forming C–H···π interactions between methyl group hydrogens and the host aromatic rings [39]. Unlike **3** and **4**, the sterically unhindered **5** sits deeper inside the cavity (Figure 2c) with *h* = 2.66 Å thereby forming numerous C–H···π interactions between protons *meta*- to the N–O group and host aromatic rings (2.86–3.0 Å).

**Table 1:** Summary of solid-state host–guest *endo*/*exo* complexations, and cavity conformation flexibility in BrC6.

Guest	*endo* */exo*	A–C (ca*.*, Å)	B–D (ca*.*, Å)	Δ [(B–D) − (A–C)]	*h*^a^ (ca*.*, Å)

**1**	NA^b^	–	–	–	–
**2**	NA^b^	–	–	–	–
**3**	*endo*	6.05	7.41	1.36	3.31
**4**	*endo*	5.84	7.54	1.70	3.29
**5**^c^	*endo*	6.24	7.33	1.09	2.66
		6.23	7.34	1.11	2.62
**6**	*endo*	5.89	7.48	1.59	3.23
**7**	*endo*	5.85	7.57	1.72	3.49
**8**	*endo*	6.25	7.33	1.08	2.82
**9**	NA^a^	–	–	–	–
**10**	–^d^	5.76	7.60	1.84	–
**11**	*endo*	5.52	7.91	2.39	4.0
**12**^c^	*endo*	5.81	7.46	1.65	2.83
		6.04	7.43	1.39	2.77

^a^*h*: Position of the *endo* cavity guest, calculated from the centroid of the lower rim host carbons to the nearest non-hydrogen atom of the guest; ^b^Crystal structure not available; ^c^Asymmetric unit contains two crystallographically independent BrC6 host molecules; ^d^self-inclusion complex.

Guests **6** and **7** have never been previously analysed by us in our earlier resorcinarene–PyNO H–G studies [39–40]. As shown in Figure 2, despite the BrC6 cavity’s flexible nature, the position of the methoxy substituent plays a crucial role for both guest orientation and the depth of the guest’s occupation of the cavity. For example, in **6**@BrC6 (Figure 2d) and **7**@BrC6 (Figure 2e), guests **6** and **7** have *h* = 3.23 Å and 3.50 Å, due to their steric demands. However, in complex **8**@BrC6 (Figure 2f) the unhindered *para*-methoxy group facilitates **8** to sit deep inside the cavity at *h* = 2.82 Å. The guest’s parallel orientation to the host aromatic rings in **6**@BrC6 is caused by either steric hindrance or unfavourable positioning. This prevents the formation of stronger C–H···π interactions; consequently, **6**@BrC6 is only stabilised by weak C–C contacts at distances of 3.31 Å. However, **7** with similar ‘*h*’ values, due to the bulky methoxy group on the core aromatic ring, is tilted towards one side with the proton *meta*- to the N–O group able to manifest C–H···π interactions with distances of 2.52 and 3.0 Å. Of all the *endo* cavity interactions, the C–H···π_(centroid)_ has the shortest contact (2.52 Å). As shown in Figure 2f, the core aromatic ring of **8** and those of BrC6 in **8**@BrC6 are parallel to each other. As a result, the bromine of the C–Br bond and the C2-position establish short contacts of 3.52 Å. However, the prominent interactions responsible for locking the H–G complex are the C–H···π (ca. 2.92 Å) and C–H···O (ca. 2.61 and 2.71 Å) contacts between guest C3 hydrogens and the host carbon/hydroxy oxygens, respectively.

From our experience, the lack of π-acidic aromatic protons in guest **10** usually results in *exo* complexes [36–37,39]. To our surprise, **10**+BrC6 forms a self-inclusion complex of BrC6 by itself as shown in Figure 2g, the property usually preferred by resorcinarenes when solvate and guest molecules are absent inside the cavity. Note that the self-inclusion complex of BrC6 has *exo* methanol solvent hydrogen bonds to host hydroxy groups. This can possibly be explained by the longer lower-rim hexyl chains providing enough intermolecular _host_(C–H)···(H–C)_host_ interactions to form a stable 3D crystal lattice. On the other hand, guest **11** with two N–O groups makes the C2-protons π-acidic enough to form an *endo* complex, **11**@BrC6 (Figure 2h). The host BrC6 undergoes a remarkable conformation change elongated to one side to accommodate the rod-shaped guest **11**. The *h* value for **11** in **11**@BrC6 is ca. 4.0 Å, which is quite high when compared to values observed for small guest molecules in BrC6 H–G complexes. However, the large Δ and *h* values are typical for rod shape guests such as **11**. Despite higher ‘*h*’ values, guest **11** is stabilised by several C–H···π interactions between C2 protons and host aromatic rings. The distances range between 2.72 and 3.0 Å, with C–H···π_(centroid)_ on two sides being the shortest contacts with distances of 2.49 Å and 2.67 Å. In our previous report, **11**+BrC2, due to the BrC2 rigid cavity the rod-shaped **11** form an *exo* complex [39]. In **12**@BrC6 (Figure 2i), the C–C bond rotation in guest **12** allows one aromatic ring to reside inside the cavity at *h* = 2.83 Å. The H–G molecules are positioned primarily by the π···π contacts rather than C–H···π interactions, with a short C···C contact being ca*.* 3.20 Å. Furthermore, since **11** is able to undergo C–C bond rotation, BrC6 tends to maintain a nearly ideal crown geometry suggesting excellent conformational complementarity between **11** and BrC6.

### Comparison of ditopic H–G complexes

**Figure 3:**
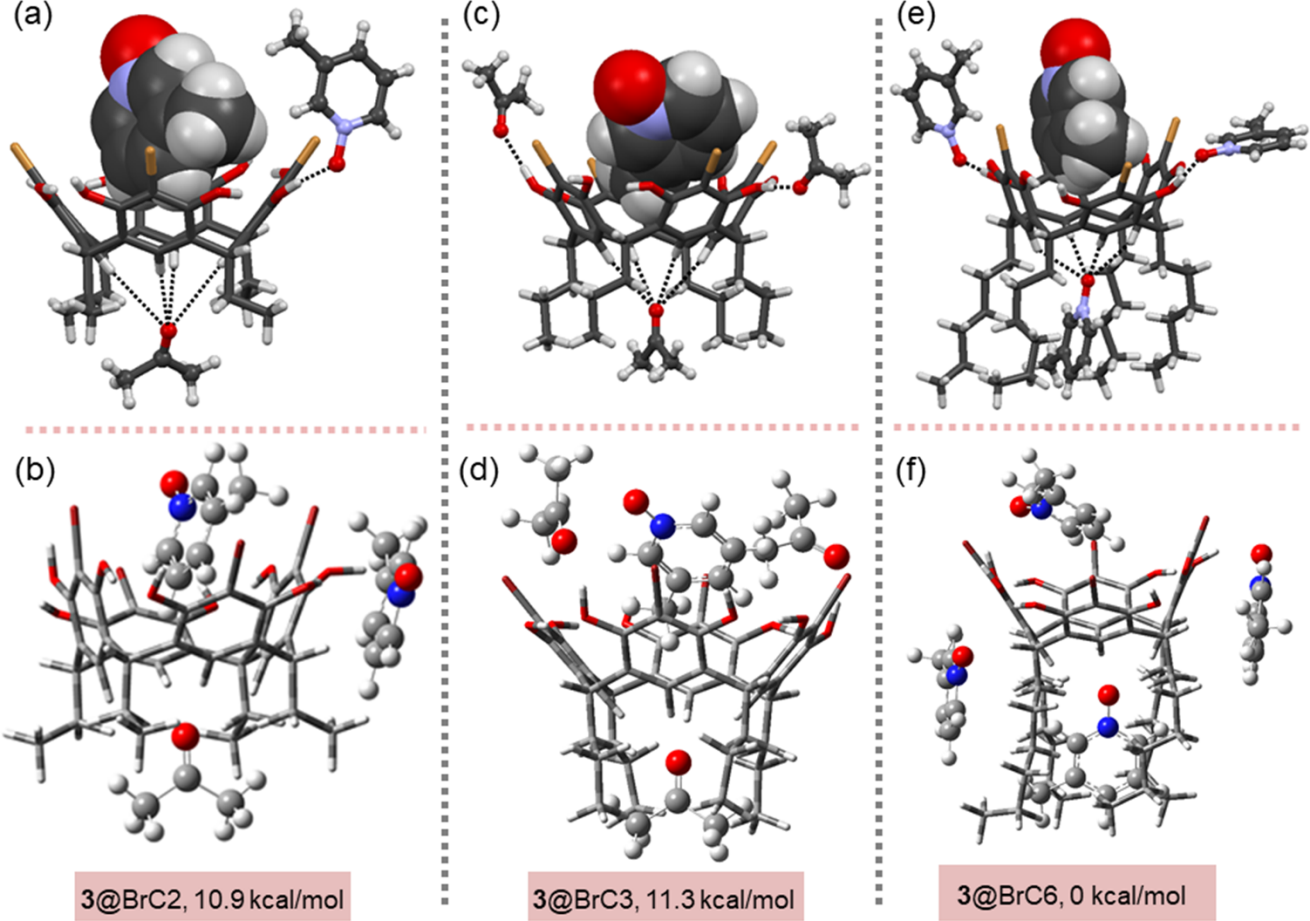
Comparison of X-ray crystal structures (a) **3**@BrC2, (c) **3**@BrC3, and (e) **3**@BrC6 and their DFT-based optimised geometries (b) **3**@BrC2, (d) **3**@BrC3, and (f) **3**@BrC6, respectively.

In **3**@BrC6, the asymmetric unit contains one host and four guest **3** molecules. Of the four guests, one resides in the upper-rim *endo* cavity, held in position by C–H···π interactions. The second sits in the lower rim between the hexyl chains and is stabilised through N–O···(H–C)_Ar(host)_ and other weak non-covalent interactions. The remaining final two guests are *exo* cavity hydrogen bonded to the host’s hydroxy groups. To our surprise, our previous X-ray crystal structures of **3**@BrC3 and **3**@BrC2 complexes obtained from acetone showed interactions with the putative guests (i.e., *N*-oxide and acetone molecules) by encapsulation within the upper-rim and lower-rim cavity [39–40]. Therefore, in an effort to better understand the host–guest interactions and the potentials of the secondary lower-rim binding mode, molecular mechanics (OPLS-2005) [42] calculations were performed on complexes, **3**@BrC2, **3**@BrC3 and **3**@BrC6 using Jaguar (Schrödinger) [43–44]. Consequently, the structures are modelled for both *exo* and *endo* complexes in acetone. Of note, the X-ray crystal structure of **3**@BrC6 (Figure 3e) is obtained from methanol and is presented here only for reference, while its corresponding computational model was optimised using acetone media. To ensure that we were adequately screening the host conformer space in these simulations, no constraints were enforced on either *N*-oxide or acetone molecules. The low energy structures obtained from these OPLS-2005 searches were then further analysed using DFT-based techniques [45–47]. The resulting optimised geometries of the **3**@BrC2, **3**@BrC3 and **3**@BrC6 along with the M06-2X/6-31G(d,p)//ωB97X-D/6-311G(d,p) calculated relative energies of complexes with respect to the most stable complex **3**@BrC6 by following isodesmic reaction schemes (see Supporting Information File 1, Table S3) are shown in Figure 3.

In the optimised structures, the inclusion complexes of **3**@BrC6, **3**@BrC3 and **3**@BrC2 show that the N–O group of **3** in **3**@BrC6, **3**@BrC3 and **3**@BrC2 is positioned outward from the host cavity similar to solid-state X-ray crystal structures as shown in Figure 3. Further, in the lower-rim, the C=O group of acetone in **3**@BrC2 and **3**@BrC3, and N–O group of **3** in **3**@BrC6 are positioned closer to the lower-rim C–H_Ar_ forming non-classical H-bond, (C–H)_Ar_···O=C/O–N, interactions. All three optimised complexes evince C–H···π interactions in both lower- and upper-rim cavities and C–H···O=C/O–N interactions at the lower-rim pocket are responsible for the ditopic behaviour of BrC2/BrC3/BrC6 and **3**. The relative energies for **3**@BrC2, **3**@BrC3 and **3**@BrC6 are 10.9, 11.3, and 0 kcal/mol, respectively, and clearly **3**@BrC6 tend to have the lowest energy and is the most stable among the three complexes. In the optimised **3**@BrC6 structure, the upper-rim *N*-oxide oxygen atom are tilted towards the hydroxy group of the host molecule to form intermolecular negative charge assisted H-bonding, C–H···O [48], interactions with a distance of 1.49 Å.

**Figure 4:**
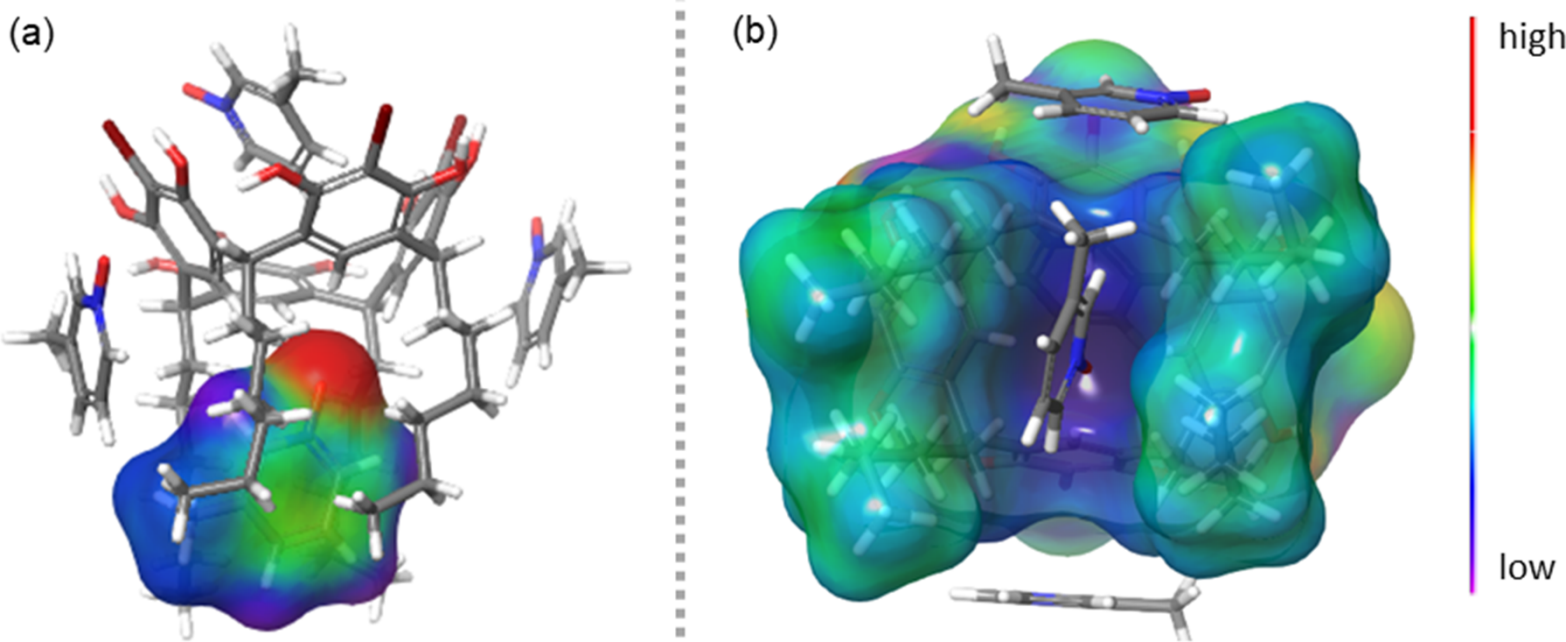
(a) The negative potential localised on the *N*-oxide oxygen in **3**@BrC6 and, (b) the positive charge distribution in lower-rim host cavity [+0.06 to −0.06 a.u.].

In order to gain insights into lower-rim cavity binding sites from a qualitative analysis standpoint, a molecular electrostatic potential (MEP) surface map for **3**@BrC6 was calculated. This shows that the host BrC6 lower-rim cavity is not neutral as might be expected, but instead contains a sharp positive electrostatic potential region as depicted with blue colour in Figure 4b. This provides an excellent opportunity for the negative potential regions of the *N*-oxide oxygen atom in guest **3** (red region in Figure 4a) to establish several intermolecular (C–H)_Ar_···O–N H-bond interactions at the lower-rim host pocket.

In addition, we used Bader’s quantum theory of atoms in molecules (QTAIM) [49] to analyse multiple non-covalent interactions (i.e., H-bonding and C–H···π) interactions in both the upper-rim *endo* cavity and the lower-rim site present in **3**@BrC6. Based on QTAIM, the presence of a bond path between the donor and the acceptor atoms containing a (3, −1) bond critical point (BCPs; highlighted as small blue circles in Figure S1, Supporting Information File 1), confirm the existence of bonds in this system. In other words, the bond critical point and bond path connecting two atoms are evidence for a real interaction rather than a simple spacial relationship. At the bond critical points, the electronic charge density [ρ(r)], and its Laplacians (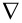
^2^ρ(r)) are important parameters to evaluate the nature and strength of interactions. Numerical values for these topological parameters related to several non-covalent interactions at both upper and lower rim of complex **3**@BrC6 are shown in Table 2 (see Supporting Information File 1, Figure S1 for the related molecular graph). Based on QTAIM analysis, the presence of several C−H···π interactions are evident from the existence of the (3, −1) bond critical point (BCPs; small red circles) between the bond path connecting the hydrogen atoms in the alkyl chain of the lower cavity in BrC6 with the *ortho, meta* and *para* carbon atoms of the *N*-oxide aromatic ring (highlighted as (C–H)_alkyl_···π_(ortho)_, (C–H)_alkyl_···π_(meta)_, (C–H)_alkyl_···π_(para)_). In addition, C–H···π interactions are present in the upper rim of the host as observed from the existence of the (3, −1) bond critical point between the bond path connecting the aromatic C−H bonds of BrC6 with *ortho, meta* and *para* carbon atoms of the *N*-oxide aromatic ring (highlighted as (C–H)_Ar_···π_(ortho)_, (C–H)_Ar_···π_(meta)_, (C–H)_Ar_···π_(para)_). The ρ(r) values associated with these interactions ranged between 0.0046 to 0.0119 a.u. and the positive values of Laplacians (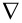
^2^ρ(r)) at the BCPs were from 0.0134 to 0.0397 a.u. suggesting the existence of a weak “closed shell” [50–52] character for non-covalent interactions (such as ionic bonds, HBs, stacking type and van der Waals interactions) between **3** and BrC6 (Table 2). This is completely consistent with the observations made from the crystal structures.

**Table 2:** Values of the density of all electrons ρ(r) and Laplacian of electron density – 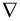
^2^ρ(r), (Hartree) at the bond critical points (3, −1) for selected significant lower-rim non-covalent C–H···π and H-bond C–H···O–N as well as upper-rim *endo* cavity C–H···π interactions in the model system **3**@BrC6 as well as calculated energies of these bonds, *E*_(x)_ (kcal/mol), proposed by Espinosa et al. [53–54].

Non-covalent motif	ρ(r)	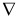 ^2^ρ(r)	*E*_(x)_^a^

Lower rim			

(C–H)_alkyl_···π_(ortho)_	0.0074	0.0247	1.2
(C–H) _alkyl_···π_(meta)_	0.0058	0.0180	1.2
(C–H) _alkyl_···π_(para)_	0.0046	0.0134	0.8
(C–H) _alkyl_···π_(para)_	0.0049	0.0156	0.8

Upper rim			

(C–H)_Ar_···π_(ortho)_	0.0090	0.0294	1.5
(C–H)_Ar_···π_(meta)_	0.0106	0.0332	1.9
(C–H)_Ar_···π_(para)_	0.0099	0.0311	1.8
C–H···O–N	0.0119	0.0397	2.9
C–H···O–N	0.0104	0.0324	10.2
C–H···O–N	0.0086	0.0268	8.4
C–H···O–N	0.0113	0.0351	11.0

^a^See Supporting Information File 1 for more details and *E*_(x)_ calculations.

### ^1^H NMR host–guest solution studies

**Figure 5:**
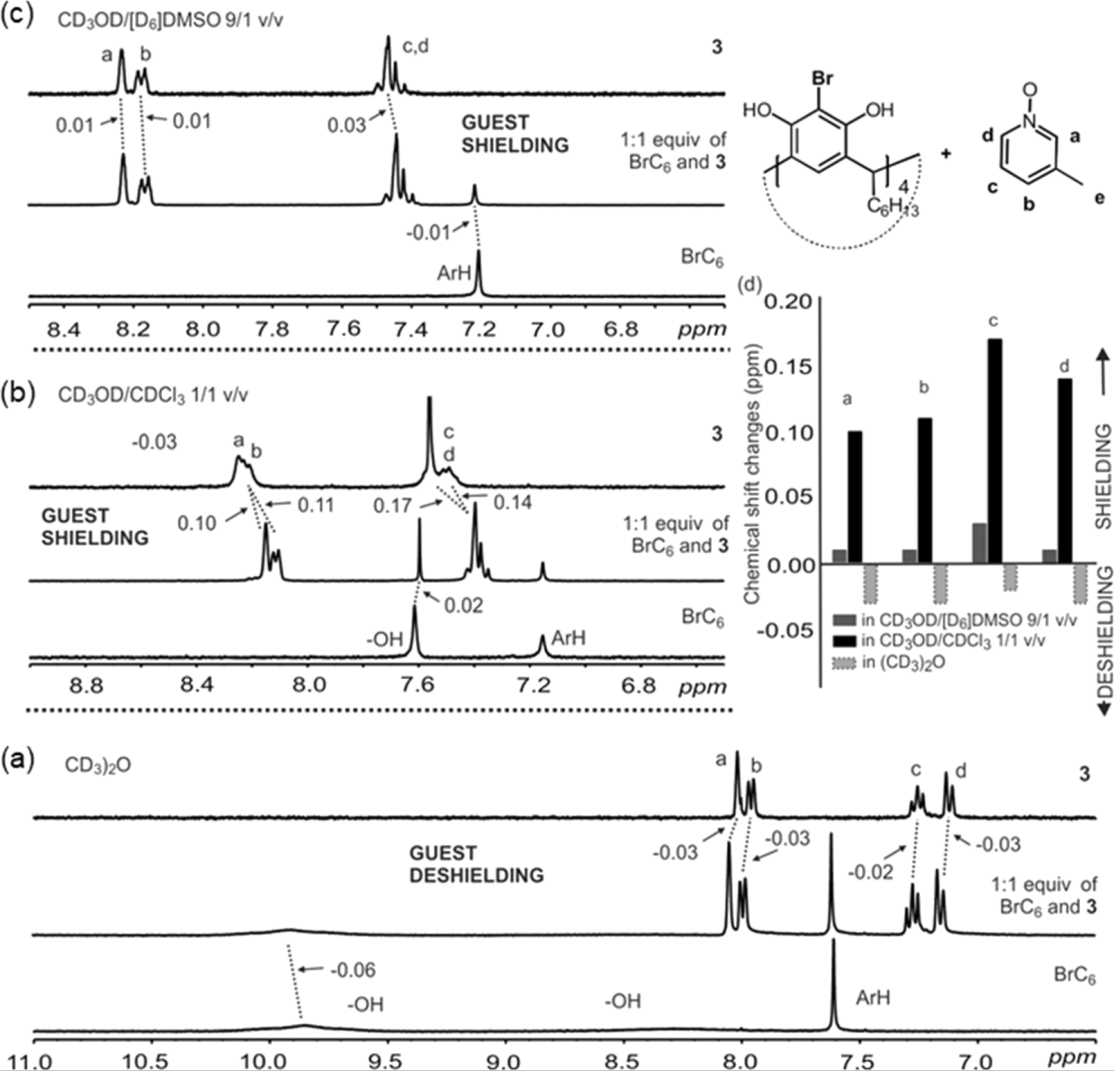
An expansion of the ^1^H NMR (6.6 mM at 298 K, 500 MHz) of BrC6 complexes with **3**. Spectra are produced from BrC6, **3** and an equimolar mixture of BrC6 and **3** in: (a) (CD_3_)_2_O, (b) CD_3_OD/CDCl_3_ 1:1 v/v, and (c) CD_3_OD/DMSO-*d*_6_ 9:1 v/v. Dashed lines highlight the observed shift changes of the resonances, labels are in ppm. (d) Bar chart showing the comparative shift changes of the guests in the different solvent media.

Guest binding studies of the *N*-oxide guests (**1**–**12**) by the receptor BrC6 were investigated in solution via a series of ^1^H NMR experiments in different hydrogen bond competing solvents and solvent mixtures: acetone-*d*_6_, methanol/chloroform (CD_3_OD/CDCl_3_) 1:1 v/v and methanol/dimethyl sulfoxide (CD_3_OD/DMSO-*d*_6_) 9:1 v/v. The above solvent mixtures were chosen due to the poor solubility of some of the guests in pure methanol. DMSO is known to be an extremely HB competitive solvent and thus prevents the clear formation of host–guest complexes [40,55], while the less competitive chloroform tends to enhance capsular assemblies [55]. Only one set of resonances from the ^1^H NMR of the receptor BrC6 in all the solvents and solvent mixtures is observed, thus confirming a symmetrical crown conformation in solution (Figure 5). Our previous report studying the interactions between BrC3 and some *N*-oxides in acetone-*d*_6_ revealed moderate deshielding of the hydroxy groups of the BrC3 receptor and minor deshielding of the aromatic protons of the guest when complexes were formed [40]. This confirmed that the assembly was driven by hydrogen bonding [55–56]. Taking the example of BrC6 and **3**, a similar moderate deshielding of the hydroxy groups of the BrC6 receptor and a minor deshielding of the aromatic protons of the guest signals are observed (Figure 5) confirming this assembly is also driven by hydrogen bonding. These shifts’ changes are substantially increased when more electron-donating groups are present on the aromatic *N*-oxides such as with **5** (two methyl groups) and **9** (two methoxy groups, Figures S5 and S9, Supporting Information File 1). This is expected as the four electron-withdrawing bromine groups on the BrC6 receptor renders the receptor slightly electron deficient further facilitating π–π interactions. With the larger *N*-oxide guests **10**–**12**, though the shift changes of the guest are not strong enough to conclusively indicate *endo* complexation, clear changes in the hydroxy groups suggest interaction via hydrogen bonding (Figures S10–S12, Supporting Information File 1).

Due to fast H/D exchange processes on the NMR time scale at 298 K in protic solvents, the hydrogen bond interactions between host and guests were not observed. In CD_3_OD/CDCl_3_, complexation-induced chemical shift changes of the guests are observed which results from the electronic shielding effects of the core aromatic rings of the host cavity. As an example, significant up-field shift changes of up to 0.17 ppm for the *c-*proton, and smaller up-field shifts of 0.10 ppm for the *a-*proton in guest **3** were observed (Figure 5b). These shifts suggest that in solution, the N–O group of guest **3** is pointing outward from the BrC6 cavity during *endo* complexation. In the X-ray structure of **3**@BrC6, only the *c*-proton of **3** has C–H···π_(host)_ short contacts with distances ranging between ca. 2.65 Å and 2.85 Å. This supports the maximum chemical shift change of 0.17 ppm observed by ^1^H NMR experiments for the *c*-proton in guest **3**. The ^1^H NMR experiments for guests **1**, **2**, and **4–9** (Figures S2–S9, Supporting Information File 1) show similar up-field chemical shift changes for the aromatic protons of *N*-oxides suggesting guests are inside the host cavity stabilised through C–H···π interactions. Very low shift changes for **11** clearly point to a minimal interaction with the host. This is contrary to the X-ray crystal structure, **11**@BrC6, where **11** and BrC6 are locked by several C–H···π interactions, and of more prominently remarkably short C–H···π_(centroid)_ interactions (2.49 Å and 2.67 Å). Interestingly, shift changes of up to 0.19 ppm for guest **12** are a clear indication for the *endo* complex. Chemical shift changes of up to 0.12 ppm for guest **10** suggest an *endo* complexation contrary to the X-ray. These observations also matches well with the presence and calculated values of energy for those interactions predicted by our computational analysis and match exactly with reported [48,53] HB interactions with medium strength as well as stacking type interactions with weak characters.

In CD_3_OD/DMSO-*d*_6_ 9:1 v/v, under similar experimental conditions to CD_3_OD/CDCl_3_ 9:1 v/v, no significant chemical shift changes were observed for nine of the twelve pyridine *N*-oxides. The above results clearly show the strong influence of DMSO in interfering with the host–guest complexation between BrC6 and the aromatic *N*-oxides. However, with guests such as **5** and **9**, *endo* cavity host–guest interactions persist even in these very competitive environments (Table 3, Figures S5 and S9, Supporting Information File 1).

**Table 3:** Summary of *endo*/*exo* host–guest complexations studied in solution by ^1^H NMR in comparison to the solid state by single crystal X-ray crystallography.

Complex	^1^H NMR solution studies			X-ray crystal structure

	(CD_3_)_2_O	CD_3_OD/ CDCl_3_ (1:1 v/v)	CD_3_OD/ DMSO-*d*_6_ (9:1 v/v)	

**1**+BrC6	–^a^	*endo*	*exo*	NA^b^
**2**+BrC6	–^a^	*endo*	*exo*	NA^b^
**3**+BrC6	–^a^	*endo*	*exo*	*endo*
**4**+BrC6	–^a^	*endo*	*exo*	*endo*
**5**+BrC6	–^a^	*endo*	*endo*	*endo*
**6**+BrC6	–^a^	*endo*	*endo*	*endo*
**7**+BrC6	–^a^	*endo*	*exo*	*endo*
**8**+BrC6	–^a^	*endo*	*exo*	*endo*
**9**+BrC6	–^a^	*endo*	*endo*	NA^b^
**10**+BrC6	–^a^	*endo*	*exo*	–^c^
**11**+BrC6	–^a^	*endo*	*exo*	*endo*
**12**+BrC6	–^a^	*endo*	*exo*	*endo*

^a^H-bonds dominate the assembly in acetone and only deshielding observed; ^b^Crystal structure not available; ^c^Self-inclusion complex.

## Conclusion

Host–guest systems formed between *C*-hexyl-2-bromoresorcinarene (BrC6) and twelve aromatic *N*-oxides have been characterised using solid-state X-ray crystallography and ^1^H NMR solution studies in three different hydrogen-bond-competitive solvents. In the solid state, BrC6 undergoes large cavity conformational changes to accommodate the *N*-oxide guests compared to our previously studied host systems, *C*-ethyl-2-bromoresorcinarene and *C*-propyl-2-bromoresorcinarene, thus proving BrC6 as more reliable host system for a range of *N*-oxide guests. In solution through ^1^H NMR analyses in methanol/chloroform, significant shielding for aromatic *N*-oxide guests suggests *endo* complexation processes similar to solid state X-ray crystal structures were observed. In methanol/DMSO-*d*_6_ chemical shift changes were observed only for three *N*-oxide guests with suitable electron-donating groups on the core aromatic ring suggesting *endo* complexation, and for other *N*-oxide guests, DMSO solvation prevents the *endo* complexation processes. In acetone-*d*_6_, significant changes for host hydroxy groups suggest host–guest assemblies were driven by hydrogen bond interactions at the upper rim. DFT based calculations using M06-2X/6-31G(d,p)//ωB97X-D/6-311G(d) support the experimental results and show that the ditopic host–guest binding modes of 3-methylpyridine *N*-oxide+BrC6 is more favourable due to longer lower-rim hexyl chains compared to 3-methylpyridine *N*-oxide+*C*-ethyl-2-bromoresorcinarene and 3-methylpyridine *N*-oxide+*C*-propyl-2-bromoresorcinarene. The predicted low energy of 3-methylpyridine *N*-oxide+BrC6 with respect to the other complexes can be attributed to multiple intermolecular hydrogen bonding and stacking interactions at both upper and lower-rims.

## Supporting Information

File 1 Experimental details, ^1^H NMR solution-data, X-ray crystallography experimental details and computational data.
