# Supplementary material for: Host–guest complexes of conformationally flexible C-hexyl-2-bromoresorcinarene and aromatic N-oxides: solid-state, solution and computational studies
Source: Beilstein J Org Chem. 2018 Jul 10;14:1723–33. doi: 10.3762/bjoc.14.146 (PMC6071688; doi:10.3762/bjoc.14.146)
Supplement: File 1 — Experimental details, 1H NMR solution-data, X-ray crystallography experimental details and computational data. [file Beilstein_J_Org_Chem-14-1723-s001.pdf]

Supporting Information

for

Host–guest complexes of conformationally flexible *C*-hexyl-2-bromoresorcinarene and aromatic *N*-oxides: solid-state, solution and computational studies

Rakesh Puttreddy[1], Ngong Kodiah Beyeh[2,3], S. Maryamdokht Taimoory[3], Daniel Meister[3], John F. Trant*[3] and Kari Rissanen[1]*

Address: [1]University of Jyvaskyla, Department of Chemistry, P. O. Box 35, 40014 Jyväskylä, Finland, [2]Department of Chemistry, Oakland University, 146 Library Drive, Rochester, Michigan 48309-4479, USA and [3]Department of Chemistry and Biochemistry, University of Windsor, 401 Sunset Avenue, Windsor, N9B 3P4, Canada

Email: John F. Trant - j.trant@uwindsor.ca, Kari Rissanen - kari.t.rissanen@jyu.fi

*Corresponding author

Experimental details, ^{1}H NMR solution-data, X-ray crystallography experimental details and computational data

Table of Contents

I General information S2

II Solid-state analyses S2

III Computational study S5

IV Solution studies S17

V References S28

I General information

All solvents used for syntheses and crystal growth are reagent grade, and are used as received. Pyridine *N*-oxide (**1**), 2-methylpyridine *N*-oxide (**2**), 3-methylpyridine *N*-oxide (**3**), 4-methylpyridine *N*-oxide (**4**), 2,6-dimethylpyridine *N*-oxide (**5**), 4-methoxypyridine *N*-oxide (**8**), 4-phenylpyridine *N*-oxide (**10**), 4,4'-bipyridine *N,N*'-dioxide (**11**) and 4,4'-bipyridine *N,N*'-dioxide (**12**) as guests were purchased from Sigma Aldrich. *C*-Ethyl-2-bromoresorcinarene (BrC2), *C*-propyl-2-bromoresorcinarene (BrC3) and *C*-hexyl-2-bromoresorcinarene (BrC6) were synthesized according to reported procedure [1]. 2-Methoxypyridine *N*-oxide (**6**), 3-methoxypyridine *N*-oxide (**7**) and 2,6-dimethoxypyridine *N*-oxide (**9**) were synthesized as reported [2]. The ^{1}H NMR spectra were recorded on a Bruker Avance DRX 500 MHz spectrometer. Chemical shifts are calibrated to the residual solvent signals.

Ia General crystallization procedure: To a solution of BrC6 (0.018 mmol) in methanol (1.0 ml), was added respective aromatic *N*-oxide (0.070 mmol) dissolved in methanol (0.5 ml) at room temperature. The reaction mixtures were heated (3-5 minutes) to clear solutions using heat-gun and the hot solutions were filtered to remove insoluble precipitates. Slow evaporation of light orange-red colour filtrates provides single crystals suitable for X-ray diffraction analysis.

II Solid-state analyses

Single-crystal X-ray data for **3**@BrC6, **4**@BrC6, **6**@BrC6, **7**@BrC6, **8**@BrC6, **11**@BrC6, and **12**@BrC6 were collected on a dual source Rigaku SuperNova Oxford diffractometer [3] equipped with an Atlas detector using mirror-monochromated Cu Kα (λ = 1.54184 Å) radiation. The data for **5**@BrC6 were measured using a Rigaku SuperNova single-source Oxford diffractometer with an Atlas EoS CCD detector using mirror-monochromated Mo-*K*α (λ = 0.71073 Å) radiation. Single-crystal X-ray data for BrC6 were measured on a Bruker-Nonius Kappa CCD diffractometer [4] equipped with an APEX-II CCD detector using graphite-monochromated Mo Kα (λ = 0.71073 Å) radiation. The data obtained from Bruker Nonius Kappa diffractometer were performed using the program COLLECT [5] and HKL DENZO AND SCALEPACK [6]. The data collection and reduction for complexes performed using Rigaku instruments were done by the program *CrysAlisPro* [3], the gaussian face index absorption correction method [3] was used for these complexes. The intensities for data collected using Bruker Nonius Kappa diffractometer were corrected for absorption using SADABS [6] with multi-scan absorption correction type method. All the structures were solved with direct methods (*SHELXS*[7]) and refined by full-matrix least squares on F^2 using the *OLEX2* software [8] which utilizes the *SHELXL*-2013 module [7]. No attempt was made to locate the hydrogens for disordered solvent molecules. Constraints (AFIX and EADP) and restraints (DFIX and ISOR) are used for disorder models in particular lower-rim alkyl chains.

Table S1: X-ray experimental details for **3**@BrC6 - **6**@BrC6

Complex	**3**@BrC6	**4**@BrC6	**5**@BrC6	**6**@BrC6
CCDC No:	1837604	1837605	1837606	1837607
Empirical formula	$C_{76}H_{96}Br_4N_4O_{12}$	$C_{61}H_{87}Br_4NO_{12}$	$C_{120}H_{161}Br_8N_2O_{20}$	$C_{60}H_{83}Br_4NO_{12}$
Formula weight	1577.20	1345.95	2590.78	1329.91
Temperature (K)	123.00(10)	120.00(10)	120.00(10)	120.00(10)
Crystal system	Triclinic	Triclinic	Triclinic	Monoclinic
Space group	*P*-1	*P*-1	*P*-1	*P2₁/c*
Unit cell dimensions: a (Å)	14.8156(5)	13.8761(5)	12.6979(8)	21.37244(18)
b (Å)	14.8686(6)	15.5316(6)	21.2707(13)	11.14313(10)
c (Å)	17.7798(6)	15.6532(6)	24.4864(15)	25.7133(2)
α (°)	88.783(3)	80.377(3)	113.785(6)	90
β (°)	71.093(3)	89.829(3)	97.433(5)	97.3173(8)
γ (°)	78.562(3)	69.999(3)	90.822(5)	90
Volume / $Å^3$	3627.8(2)	3120.1(2)	5984.9(7)	6073.90(9)
Z	2	2	2	4
Density (calculated) mg/m^3	1.444	1.433	1.438	1.454
Absorption Coefficient mm^{-1}	3.221	3.626	2.746	3.719
F(000)	1632	1392	2670	2744
Crystal size (mm^3)	0.13 x 0.12 x 0.05	0.10 x 0.08 x 0.05	0.16 x 0.10 x 0.07	0.25 x 0.17 x 0.05
θ range for data collection (°)	3.04 to 66.75	3.40 to 66.75	2.99 to 25.0	3.47 to 66.75
Reflections collected	21600	17730	53033	33131
[R(int)]	[0.0327]	[0.0371]	[0.1019]	[0.0358]
Reflections [I>2sigma(I)]	11180	8958	11813	9966
Data completeness (%)	99.04	98.97	99.82	99.83
Data/ restraints/ parameters	12752/0/889	10953/44/712	21041/45/1343	10761/0/705
Goodness-of-fit on F^2	1.119	1.028	1.048	1.026
Final R_1 indices [I>2sigma(I)]	R_1 = 0.0368, wR_2 = 0.0861	R_1 = 0.0590, wR_2 = 0.1547	R_1 = 0.0940, wR_2 = 0.2253	R_1 = 0.0361, wR_2 = 0.0952
Final R indices [all data]	R_1 = 0.0433, wR_2 = 0.0893	R_1 = 0.0722, wR_2 = 0.1658	R_1 = 0.1545, wR_2 = 0.2732	R_1 = 0.0383, wR_2 = 0.0980
Largest diff. peak/hole (e.$Å^{-3}$)	0.556/ -0.583	1.675/ -2.546	3.676/ -0.970	1.243/ -0.552

Table S2. X-Ray Experimental details for **7**@BrC6 to **11**@BrC6

Complex	**7**@BrC6	**8**@BrC6	MeOH+BrC6	**11**@BrC6	**12**@BrC6
CCDC No:	1837608	1837609	1837610	1837611	1837612
Empirical formula	$C_{130}H_{172}Br_8N_4O_{26}$	$C_{65}H_{86}Br_4N_2O_{13}$	$C_{53}H_{72}Br_4O_9$	$C_{65}H_{88}Br_4N_2O_{13}$	$C_{64}H_{84}Br_4N_2O_{12}$
Formula weight	2845.99	1422.99	1172.74	1425.01	1392.97
Temperature (K)	120.01(10)	120.00(1)	170.0(1)	120.0(1)	120.0(1)
Crystal system	Triclinic	Triclinic	Triclinic	Triclinic	Triclinic
Space group	*P*-1	*P*-1	*P*-1	*P*-1	*P*-1
Unit cell dimensions: a (Å)	10.7228(11)	11.9096(2)	10.501(2)	10.0437(3)	15.2700(9)
b (Å)	14.9600(15)	13.4509(2)	15.161(3)	17.0384(4)	18.0854(7)
c (Å)	21.203(2)	20.6387(4)	17.053(3)	21.0991(7)	24.1760(12)
α (°)	103.231(9)	91.3963(15)	90.81(3)	69.181(3)	75.629(4)
β (°)	90.560(8)	101.1181(17)	107.46(3)	76.589(3)	88.489(4)
γ (°)	104.941(9)	90.7080(14)	94.12(3)	79.976(2)	83.697(4)
Volume / $Å^3$	3190.4(6)	3242.71(11)	2581.3(10)	3266.16(18)	6428.5(6)
Z	1	2	2	2	4
Density (calculated) mg/m^3	1.481	1.457	1.509	1.449	1.439
Absorption Coefficient mm^{-1}	3.598	3.540	3.173	3.515	3.547
F(000)	1468	1468	1204	1472	2872
Crystal size (mm^3)	0.06 x 0.06 x 0.04	0.33 x 0.26 x 0.17	0.28 x 0.22 x 0.21	0.09 x 0.07 x 0.04	0.18 x 0.06 x 0.04
θ range for data collection (°)	3.36 to 66.75	3.29 to 66.75	2.53 to 25.25	4.14 to 66.75	2.91 to 66.75
Reflections collected [R(int)]	18440 [0.0989]	19340 [0.0227]	11117 [0.0404]	18594 [0.0407]	37439 [0.0618]
Reflections [I>2sigma(I)]	6912	10571	22255	9439	16443
Data completeness (%)	98.81	99.08	99.50	99.08	98.97
Data/ restraints/ parameters	11163/59/793	11378/7/793	9313/0/605	11474/11/824	22566/104/1550
Goodness-of-fit on F^2	1.002	1.037	1.011	0.976	1.031
Final R_1 indices [I>2sigma(I)]	R_1 = 0.0736, wR_2 = 0.1672	R_1 = 0.0289, wR_2 = 0.0753	R_1 = 0.0397, wR_2 = 0.0803	R_1 = 0.0468, wR_2 = 0.1171	R_1 = 0.0891, wR_2 = 0.2289
Final R indices [all data]	R_1 = 0.1217, wR_2 = 0.2041	R_1 = 0.0316, wR_2 = 0.0776	R_1 = 0.0627, wR_2 = 0.0882	R_1 = 0.0567, wR_2 = 0.1221	R_1 = 0.1142, wR_2 = 0.2529
Largest diff. peak/hole (e.$Å^{-3}$)	0.993/ -1.669	0.595/ -0.531	0.474/ -0.487	0.990/ -0.620	3.604/ -1.372

III Computational study

IIIa General Information

Molecular mechanics analysis of the complexes between three C-hexyl-2-bromoresorcinarene (BrC6), *C*-ethyl-2-bromoresorcinarene (BrC2) and *C*-propyl-2-bromoresorcinarene (BrC3) hosts and *N*-oxide **3** were initially carried out using Jaguar/Maestro software package [9] and OPLS-2005 force field. In order to make sure that we were adequately screening the conformer space of the complexes in these simulations, no constraints were applied on either *N*-oxide or acetone molecules.

The low energy conformer of **3**@BrC2, **3**@BrC3, and **3**@BrC6 complexes were then optimized using the Gaussian 09 suite [10] of programs at the density functional theory (DFT) level with M062X/6-31G(d,p) [11] within the IEF-PCM solvation model [12]. All of the optimized complex geometry were confirmed by frequency calculations as minima with zero imaginary frequencies. Single point calculations were performed on these optimized structures using long-range corrected (LRC) exchange-correlation functional with inclusion of dispersion correction, ωB97X-D in order to obtain a more accurate treatment of stacking type interactions [13].

Structure analysis such as Molecular Electrostatic Potential (MEP) surface map, was performed using GaussView v5.0.8.4.

A topological analysis of the electron density was performed with Bader's quantum theory of atoms in molecules (QTAIM) using the AIM2000 software [14].

Of note, the energies implemented in Table 2 are not interaction energies. We believe that calculated and predicted interaction energy for more than three components (out *N*-oxide, in *N*-oxide, as well as acetone molecule with receptor) won't be much accurate due to basis set superposition errors" (BSSEs) and "basis set incompleteness errors" (BSIEs) and in our idea the counterpoise correction of interaction energy for removing these errors won't be effective to completely remove the errors. The reported energies in Table 2, obtained from quantum theory of atom in molecule (QTAIM) only shows the contributions of different possible non-covalent interactions on energetic aspect and stability of the calculated structures and provide a basis to explain the presence of these attractive interactions in the systems and distinguish them from weak interactions. As mentioned in Table 2 (See manuscript), electron density p(r) and Laplacian of the electron density $\nabla^2 p(r)$ at the BCP, is related to bond order and in turn bond strength. $E_{(X)}$ is the energy [15] of those bonds (vary from 2.9 to 11.0 kcal/mol for H-bonds and 0.8 to 1.9 kcal/mol for other classes of non-covalent interactions) which is calculated from following equation. Although, they have important role in energetic aspect of complex, they are not the interaction energy.

$E_{(X)} = 1/2V_c$

$V_c = 1/4\ \nabla^2 p(r_c) - 2G_c$

Where Vc is the potential energy density and the kinetic energy density at the BCP.

Table S3: Isodesmic reaction schemes for comparing relative energy of the **3**@BrC2, **3**@BrC3 and **3**@BrC6 complexes.

	Isodesmic Reaction Scheme	Relative Energy (kcal/mol)
**3@BrC2**	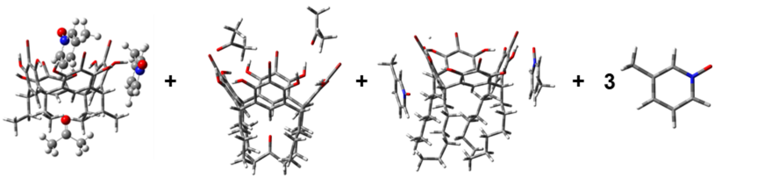	11.3
**3@BrC3**	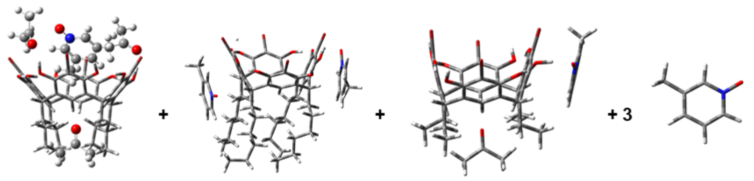	10.9
**3@BrC6**	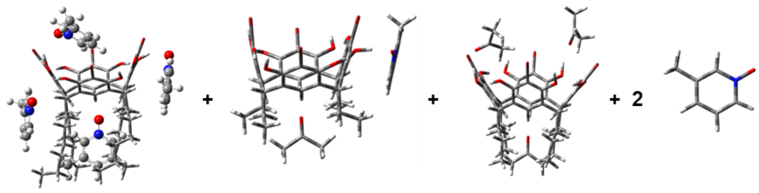	0

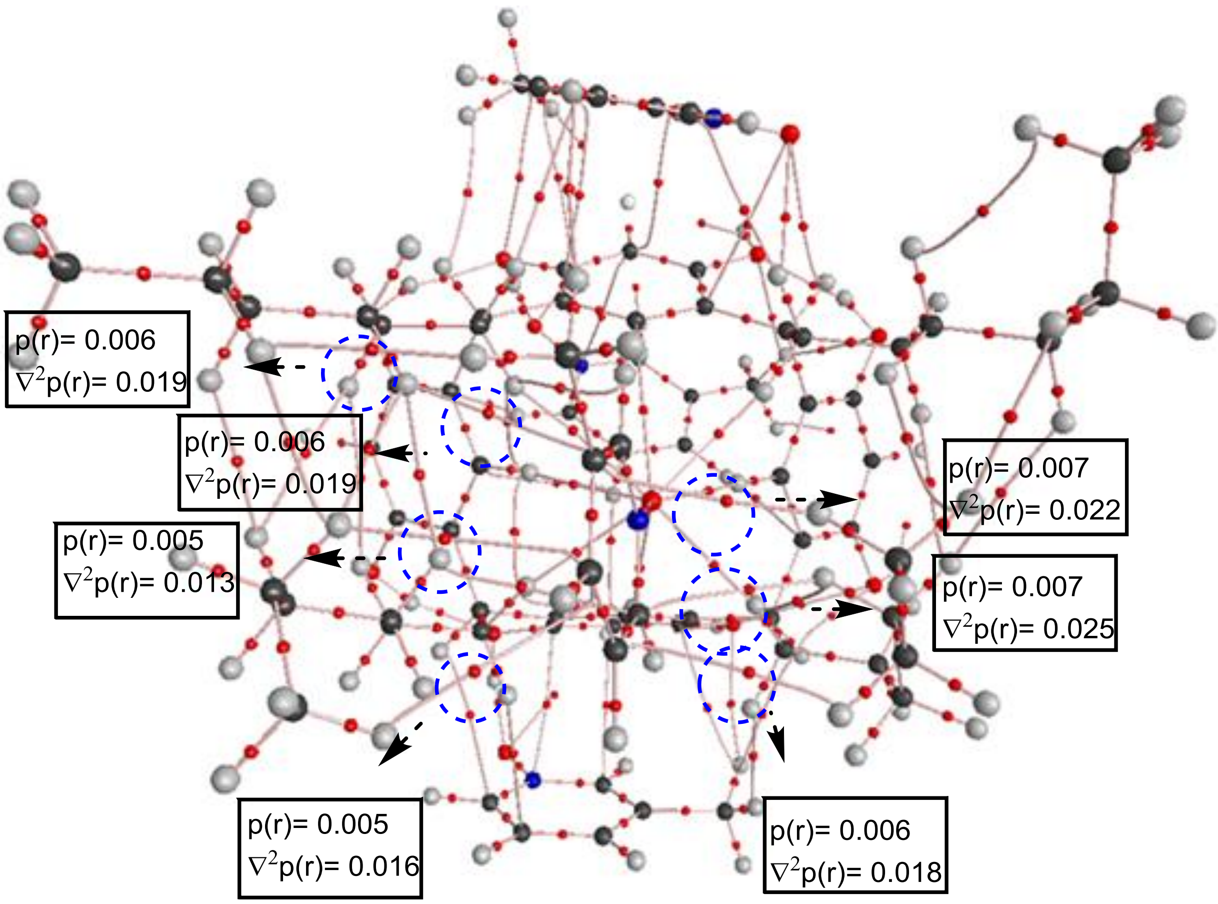

Figure S1: The plotted molecular graph and topological properties **3**@BrC6 complex by QTAIM analysis.

IIIb. DFT Calculated host-guest complex geometries for 3@BrC2

Br	-0.21242300	3.75438200	-2.27278600
Br	-6.05212000	-0.43967100	-1.67032500
Br	0.03492100	-3.44449800	-3.56893200
Br	6.19317600	-0.54449200	-1.54851100
O	2.20394000	2.85179800	-0.80239700
H	3.02694900	2.49342600	-0.42903500
O	-2.50197400	3.13352500	-0.39352500
H	-2.48347900	4.16368700	-0.25666000
O	-4.70569500	1.68856800	-0.05979300
H	-3.95255100	2.32555500	-0.12399100
O	-4.77586200	-3.04689100	-0.64772600
H	-5.44182900	-2.78836000	-1.30528500
O	-2.33428500	-3.59395400	-1.81293500
H	-3.16527400	-3.63239200	-1.30935300
O	2.39436600	-3.73305300	-1.64299800
H	2.26176900	-3.70064600	-2.60374300
O	4.87404800	-3.07835700	-0.64998100
H	4.17786100	-3.67232500	-0.97686700
O	4.67414700	1.65010800	-0.00770600
H	5.37753600	1.61263400	-0.67537100
C	-0.04870700	1.50024000	1.76357200

H	-0.01433400	0.92094400	2.68191200
C	1.15468600	1.83477800	1.14496100
C	1.10977700	2.50687500	-0.08813800
C	-0.13335800	2.87595300	-0.60838800
C	-1.32207200	2.63704600	0.08463200
C	-1.28955500	1.89985600	1.27455300
C	-2.59714000	1.52508300	1.95538400
H	-3.28897600	2.36533100	1.82362100
C	-2.47728400	1.28144200	3.46820400
H	-3.46455300	0.97356900	3.82944300
H	-1.80592300	0.43843600	3.66534800
C	-2.00962300	2.51037800	4.24227700
H	-2.69447900	3.35111300	4.08712600
H	-1.00846100	2.82291600	3.92610000
H	-1.97370500	2.30593500	5.31574200
C	-2.69710000	-0.96338800	1.47702500
H	-1.85111700	-1.08068300	2.15359800
C	-3.20818900	-2.10242100	0.85651700
C	-4.26653100	-1.93398900	-0.04107900
C	-4.73419300	-0.65339800	-0.33506000
C	-4.21033700	0.48195200	0.28892500
C	-3.19544300	0.31798300	1.24638200
C	-2.59173700	-3.47781300	1.09882200
H	-3.26757600	-4.22739900	0.66762500
C	-2.50206800	-3.79998400	2.59794100
H	-1.98794300	-4.76157700	2.71483800
H	-1.88390500	-3.04861100	3.10237300
C	-3.87695600	-3.85648600	3.25609200
H	-4.38593900	-2.89132400	3.17815800
H	-3.79145400	-4.10965000	4.31566800
H	-4.50996400	-4.61024100	2.77727200
C	-0.03025100	-3.56810100	1.04463900
H	-0.03927700	-3.46504000	2.12445500
C	1.20238500	-3.64159200	0.39754400
C	1.19591200	-3.69980100	-1.00006800
C	-0.00721700	-3.66175800	-1.69549500
C	-1.23586300	-3.62662800	-1.03306800
C	-1.25444900	-3.58219300	0.37084600
C	2.54765100	-3.52127600	1.10189700
H	3.21052800	-4.29027800	0.68730600
C	2.49288600	-3.76472800	2.61829900
H	3.48846400	-3.54686400	3.01936200
H	1.81142400	-3.04300000	3.08382300
C	2.09639600	-5.19203100	2.98881800
H	2.10254500	-5.32624100	4.07389000
H	2.79880100	-5.91189500	2.55730100
H	1.09693900	-5.44388800	2.62270300
C	2.60068100	-1.00234000	1.34199500
H	1.71999900	-1.09959800	1.97856400
C	3.11593500	0.27190900	1.11467900
C	4.21162400	0.39375700	0.25695600
C	4.76830300	-0.74466700	-0.32572300
C	4.26005300	-2.01978000	-0.06451700
C	3.15443400	-2.15668600	0.78928800
C	2.49814400	1.49795600	1.77159600
H	3.16631700	2.34922500	1.58966000
C	2.43631400	1.33090000	3.29908900

H	1.90075500	2.19149300	3.71931600
H	1.85032700	0.44023400	3.55221400
C	3.82492300	1.22099000	3.92095100
H	3.75887500	1.13076200	5.00806100
H	4.42881700	2.10409700	3.68925200
H	4.35433500	0.34244500	3.54026900
O	-2.31010100	5.58218400	0.19013500
N	-1.06162500	5.63992200	0.62289500
C	-0.75755000	5.14866400	1.84543400
H	-1.60700000	4.79695800	2.41563900
C	0.55852600	5.08471000	2.25445600
H	0.78360700	4.67820600	3.23347800
C	1.56893700	5.51938500	1.39840600
H	2.61023900	5.43370100	1.69208600
C	1.23920600	6.05550900	0.15648700
C	-0.10690200	6.13422000	-0.18403900
H	-0.47076500	6.51215100	-1.12991000
C	2.26814600	6.46088100	-0.85936600
H	2.06423700	7.45662500	-1.25990100
H	2.23754000	5.74723200	-1.68916700
H	3.26985900	6.45244500	-0.42797500
O	2.22061100	0.38337700	-3.75850100
N	1.16519100	0.18681100	-3.04798000
C	-0.04807900	0.59794600	-3.49965900
H	-0.01011200	1.10728000	-4.45394100
C	-1.21025000	0.40586200	-2.76781500
C	-1.12220600	-0.25747300	-1.54539200
H	-2.01709200	-0.44329200	-0.96246100
C	0.12595200	-0.64036200	-1.07341300
H	0.23506600	-1.12636100	-0.10885300
C	1.25680400	-0.42438800	-1.83774100
H	2.26323600	-0.70980000	-1.55433100
C	-2.51340300	0.98853600	-3.24018800
H	-3.36525200	0.41891100	-2.85655000
H	-2.57338200	1.02543900	-4.33019500
H	-2.59358400	2.01247900	-2.85639000
O	0.00079600	-1.36409500	3.61500300
C	-0.02512200	-0.38217300	5.78702000
H	0.74441300	-0.49110000	6.55611500
H	-0.99517100	-0.38733300	6.29665000
H	0.09906400	0.56455800	5.25802700
C	0.02305400	-1.53941000	4.82371700
C	0.10006200	-2.92330500	5.41399000
H	-0.63660400	-3.04195600	6.21315100
H	1.08997600	-3.06144000	5.86286200
H	-0.05424800	-3.67937600	4.64181700

IIIc. DFT Calculated host-guest complex geometries for 3@BrC3

Br	2.46935800	5.12096800	0.97065900
Br	-4.78850000	2.64165900	1.02130200
Br	-3.19170800	-2.79471700	-4.15443100
Br	4.00981400	0.13405400	-4.25348700
O	2.26120200	-2.35097000	-3.78519200
H	2.58497200	-1.84472500	-4.54671000
O	-0.29597900	-3.46035900	-3.82644700
H	0.63444000	-3.17412200	-3.84563900
O	-4.18807800	-2.45270700	-1.37424500
H	-4.44165000	-1.83394000	-0.66100800
O	-4.62905300	-0.40843400	0.35844700
H	-5.18308400	0.27483300	-0.10778900
O	-2.28505400	3.09492900	2.71310700
H	-3.01464500	3.62776100	2.35868000
O	0.31320500	4.06857900	2.71914100
H	-0.60073900	3.74273300	2.81913600
O	4.34616500	2.69882000	0.58654800
H	4.27092100	3.51895300	0.04182500
O	4.72000000	1.02320700	-1.51732100
H	4.72116500	1.54968700	-0.69184300
C	-1.56598300	-0.43369700	2.29880700
H	-0.81444000	-1.15540100	2.60954600
C	-1.42689400	0.88974300	2.71183600
C	-2.40226500	1.79907400	2.30030100
C	-3.47106900	1.37796100	1.50583900
C	-3.59564000	0.04276000	1.10942300
C	-2.63685300	-0.88939100	1.53510000
C	-2.78934600	-2.36631300	1.18111100
H	-3.86143800	-2.56054000	1.06486500
C	-2.11204300	-2.67975600	-0.14637100
C	-2.85036600	-2.66479000	-1.34014700
C	-2.19841900	-2.90920400	-2.55162500
C	-0.83438900	-3.21228300	-2.60786800
C	-0.09661300	-3.26859600	-1.41364500
C	-0.75219100	-2.97618900	-0.21836600
H	-0.17097100	-2.97604900	0.70204100
C	1.38886400	-3.60470200	-1.42753300
H	1.60139200	-4.13155300	-2.36518800
C	2.24435000	-2.34117200	-1.41700400
C	2.65969200	-1.76495600	-2.62074900
C	3.46408900	-0.62655600	-2.60812700
C	3.89481500	-0.04063000	-1.41288100
C	3.48370200	-0.60450100	-0.19365700
C	2.65061200	-1.72313100	-0.23670400
H	2.31946600	-2.14770500	0.70777900
C	3.95460600	-0.02139500	1.13828700
H	4.92543100	0.45616100	0.96409100
C	2.99131700	1.05706200	1.62329000
C	1.84774700	0.75337500	2.35991500
H	1.64823200	-0.28766000	2.60710500
C	0.94126000	1.72247800	2.79290200
C	1.15631500	3.05485300	2.41263700
C	2.28835100	3.37197500	1.65382400
C	3.21715000	2.39792900	1.28591200
C	-0.28216600	1.34003300	3.61263000

H	-0.61792500	2.23765800	4.14464500
C	0.03602600	0.29900300	4.70077500
H	0.28876000	-0.66863500	4.24878300
H	0.93281000	0.62562400	5.24134600
C	-1.11889600	0.11561200	5.68438500
H	-1.25437200	1.04331100	6.25365900
H	-2.05024000	-0.04500500	5.12764000
C	-0.88749100	-1.05572700	6.63336500
H	-1.68428000	-1.13633200	7.37718700
H	0.06393600	-0.95105900	7.16560800
H	-0.85792200	-1.99778900	6.07466400
C	-2.31444500	-3.30370800	2.30414600
H	-2.43980800	-4.33402000	1.94739700
H	-1.24249000	-3.17348400	2.48815700
C	-3.06600400	-3.12915600	3.62178200
H	-4.14199000	-3.25880600	3.45077700
H	-2.93031700	-2.10541900	3.99169300
C	-2.58200400	-4.12031400	4.67682400
H	-3.10388100	-3.98604200	5.62787300
H	-1.50991900	-3.98929900	4.86365900
H	-2.73426500	-5.15301800	4.34690900
C	1.78886300	-4.57226100	-0.30006000
H	1.68944900	-4.08575500	0.67810100
H	1.08158400	-5.41050700	-0.29455000
C	3.21403600	-5.10100600	-0.46315900
H	3.25399700	-5.76159000	-1.33767900
H	3.89139100	-4.26503700	-0.67587500
C	3.70105500	-5.83913900	0.77927400
H	3.75430200	-5.15113400	1.63100300
H	4.69742100	-6.26412000	0.63279800
H	3.02120000	-6.65468800	1.04833500
C	4.20036500	-1.09046000	2.21560300
H	4.51459700	-0.56854500	3.12860100
H	3.26786700	-1.60730700	2.46675200
C	5.25507400	-2.12876200	1.83971000
H	4.92401100	-2.68915100	0.95655000
H	6.18579300	-1.62149500	1.55617500
C	5.51948900	-3.09709800	2.98945300
H	4.59744700	-3.61395000	3.27919500
H	5.89037700	-2.56735200	3.87271900
H	6.25471600	-3.85799200	2.71471900
O	-3.95349300	2.84094200	-2.11890000
N	-2.79960300	2.72478500	-1.55778500
C	-2.27356600	3.76560900	-0.86242300
H	-2.90525800	4.64224300	-0.83311600
C	-1.02367200	3.66570500	-0.27512800
H	-0.61410600	4.51613100	0.26051000
C	-0.30406800	2.48394600	-0.38824800
H	0.68323600	2.38203200	0.04489300
C	-0.85412900	1.40748700	-1.08380100
C	-2.10782900	1.55787100	-1.65129300
H	-2.61088500	0.78246100	-2.21594600
C	-0.09616700	0.12121300	-1.21004600
H	-0.62278500	-0.60130100	-1.83724400
H	0.04981500	-0.32721600	-0.22155200
H	0.89941000	0.30079800	-1.63294600
O	4.53109100	4.21862100	-1.56838900

C	4.20835300	4.02432400	-3.91334600
H	5.05181400	4.70954600	-3.98532000
H	3.35406800	4.39753300	-4.48451700
H	4.48017600	3.05092300	-4.33602400
C	3.82707100	3.80358000	-2.47744300
C	2.53867900	3.06524500	-2.22397400
H	2.41230400	2.25728300	-2.95072700
H	1.70602600	3.76794900	-2.34421500
H	2.51116200	2.66082500	-1.21143300
O	1.09660500	-2.61170400	2.72781800
C	2.08409200	-3.00216900	4.85994800
H	2.49677000	-1.99781300	4.75009700
H	2.87059400	-3.71569300	5.12050800
H	1.36162700	-3.00648900	5.68442500
C	1.37404100	-3.42236500	3.60061800
C	1.02192300	-4.87887500	3.45636700
H	1.93968600	-5.43504600	3.23216200
H	0.30848700	-5.02312200	2.64322000
H	0.62528300	-5.27930400	4.39301500
O	-6.27427200	1.00906900	-1.15044000
C	-6.72960400	2.25520600	-3.11687600
H	-7.19961000	1.85886100	-4.02106100
H	-5.95308000	2.96292800	-3.42246800
H	-7.46684500	2.75944100	-2.49291100
C	-6.05588200	1.15592200	-2.34648000
C	-5.16611200	0.22642100	-3.12192900
H	-4.57535300	-0.40302700	-2.45688800
H	-4.52103800	0.80069900	-3.78976400
H	-5.79502800	-0.42682500	-3.73757500

IIIc. DFT Calculated host-guest complex geometries for 3@BrC6

Br	-6.03679900	-1.83405900	-0.14046300
Br	-4.15722300	5.56463300	-0.97836900
Br	-0.51799800	2.87327800	5.32892700
Br	-1.03029900	-4.93054200	4.54504800
O	-4.89634600	0.48223500	-1.81862500
H	-5.73665900	0.25688300	-1.39125000
O	-3.66637200	-3.73981700	0.11814300
H	-4.40259900	-4.22031700	-0.43554400
O	-4.15611100	3.13027100	-2.86499900
H	-4.70291400	3.91298200	-2.70155300
O	-1.50888300	5.41803900	0.35753100
H	-0.96108500	5.18123700	1.12649600
O	-0.42880200	4.22952200	2.59171300
H	-0.77602300	4.42716100	3.47582400
O	0.47220500	0.03745600	4.72671300
H	-0.54175900	0.03797900	4.95040500
O	0.95233800	-2.67445800	4.65181900
H	1.00055000	-1.71227100	4.82295100
O	-1.85555300	-5.05011000	1.65325700
H	-2.52945000	-4.73236900	1.01474300
C	-2.69831600	-0.32785800	-2.09871800
C	-1.74123800	-1.29762700	-1.80053500
H	-0.73016800	-1.15143600	-2.16775800
C	-2.02377600	-2.43513200	-1.04036900
C	-3.32683700	-2.60766000	-0.55557800
C	-4.27791300	-1.61231100	-0.79376600
C	-3.98168700	-0.47961500	-1.55666700
C	-2.40144800	0.88235300	-2.97209800
H	-3.30222800	1.07998500	-3.56327200
C	-1.23624800	0.63439700	-3.93980500
H	-0.30391200	0.53263300	-3.37120300
H	-1.39641300	-0.32654700	-4.45020300
C	-1.08984700	1.74005100	-4.98187600
H	-1.01032700	2.71240800	-4.47624300
H	-2.00177300	1.77775900	-5.59103200
C	0.13200100	1.53091000	-5.87790900
H	0.13938000	0.49019000	-6.23401500
H	0.05392200	2.15969400	-6.77293700
C	1.46289600	1.83078500	-5.18669700
H	1.53170700	2.90927600	-4.98234000
H	1.50720700	1.33723800	-4.20345900
C	2.66151500	1.39240900	-6.02412200
H	2.59046700	0.31209300	-6.20639600
H	2.60969600	1.87400100	-7.00902800
C	3.99303800	1.71614600	-5.35802100
H	4.84240000	1.34085700	-5.93622100
H	4.04271900	1.26759600	-4.35902300
H	4.11214600	2.79853700	-5.24237900
C	-2.14720500	2.11296200	-2.11214200
C	-3.07053800	3.16783800	-2.06041100
C	-2.83505100	4.22498200	-1.17575800
C	-1.65832600	4.32229300	-0.42702100
C	-0.68762400	3.31728700	-0.54554500
C	-0.98436000	2.21698700	-1.35145800

H	-0.25957500	1.40277600	-1.39911100
C	0.65952400	3.42358800	0.15946700
H	0.78731500	4.44914900	0.52073800
C	1.83652300	3.18175900	-0.79548400
H	2.74583000	3.19990100	-0.18270500
H	1.77188400	2.18914900	-1.25964400
C	1.94972000	4.24849300	-1.88157900
H	1.16214600	4.10429500	-2.63443300
H	1.78107100	5.24087100	-1.44013700
C	3.32374900	4.23277500	-2.54766800
H	4.07957000	4.43202700	-1.77600900
H	3.53405700	3.22340100	-2.93123400
C	3.46375700	5.24435200	-3.68448200
H	3.07505600	6.21768300	-3.35337200
H	2.82515500	4.92884900	-4.52019000
C	4.90380100	5.42819900	-4.17493400
H	5.34858800	4.44503900	-4.37642800
H	4.89365300	5.96377800	-5.13087800
C	5.78608500	6.19187200	-3.18786300
H	6.79762600	6.32223500	-3.58169700
H	5.86952900	5.67081600	-2.22977000
H	5.37156600	7.18569600	-2.99017700
C	0.70785300	2.53076600	1.38430200
C	0.12352400	2.98256700	2.56962200
C	0.12897000	2.16949700	3.70023900
C	0.64250100	0.87170600	3.66596400
C	1.31908200	0.44686000	2.51760600
C	1.32599700	1.28137800	1.39876200
H	1.78859600	0.92436900	0.48194500
C	1.96561900	-0.92586500	2.52778600
H	2.41882700	-1.06175700	3.51761600
C	3.10143400	-1.06790100	1.50401100
H	2.69413700	-1.06587400	0.48390100
H	3.74773100	-0.18433800	1.58787100
C	3.94026000	-2.32729800	1.71447900
H	4.24112300	-2.38885000	2.76997700
H	3.33190900	-3.21994400	1.51125400
C	5.19253200	-2.34209800	0.84015700
H	4.89691200	-2.33031700	-0.21992900
H	5.75618000	-1.41282400	1.01274800
C	6.10821500	-3.53573200	1.10400700
H	5.58864800	-4.46551800	0.83274500
H	6.31109400	-3.60199400	2.18209300
C	7.43644200	-3.46284700	0.35480000
H	7.24321300	-3.39497900	-0.72319900
H	7.95815500	-2.53862000	0.63533300
C	8.33006100	-4.66870400	0.63206200
H	9.28097800	-4.59797100	0.09763600
H	7.83696600	-5.59580400	0.32104000
H	8.54927100	-4.75321300	1.70127300
C	0.91352200	-2.00451000	2.33398100
C	0.48780900	-2.82761800	3.38609400
C	-0.44653200	-3.83539100	3.12306700
C	-0.96594100	-4.04616000	1.83955900
C	-0.53810400	-3.22078100	0.78733300
C	0.35748600	-2.19524200	1.07341300
H	0.66013200	-1.52556700	0.26737500

C	-0.96712100	-3.45634300	-0.65822900
H	-1.42367200	-4.45233200	-0.71837400
C	0.25299900	-3.44165500	-1.59353000
H	-0.09445400	-3.57522600	-2.62712400
H	0.72970400	-2.45464400	-1.55325300
C	1.30196500	-4.49768900	-1.24408300
H	0.94572000	-5.49569800	-1.53190300
H	1.44688800	-4.52056900	-0.15617700
C	2.64687400	-4.20731500	-1.90506500
H	2.52731400	-4.20198500	-2.99663700
H	2.96177600	-3.18781700	-1.62530000
C	3.75132900	-5.17632400	-1.49397200
H	3.46984800	-6.20229700	-1.76932100
H	3.83254700	-5.16046700	-0.39874200
C	5.10953800	-4.83608700	-2.11082500
H	5.33445100	-3.77892200	-1.90633600
H	5.89459400	-5.41915900	-1.61286200
C	5.17479600	-5.08287400	-3.61669500
H	6.16610300	-4.84688800	-4.01389300
H	4.44792900	-4.46682200	-4.15501500
H	4.95943200	-6.13120800	-3.84772700
O	-5.22073700	-4.76116200	-1.55535600
N	-4.47647000	-4.40844300	-2.58859300
C	-3.37206800	-5.12037300	-2.89138600
H	-3.21135900	-5.99688200	-2.27786300
C	-2.53690800	-4.69442900	-3.90894100
H	-1.65062800	-5.27529000	-4.13443200
C	-2.83205600	-3.52539900	-4.60200200
H	-2.16576200	-3.16737700	-5.38035700
C	-3.98883800	-2.80601300	-4.28965700
C	-4.80111700	-3.29478000	-3.27776100
H	-5.69868000	-2.80128200	-2.92309000
C	-4.36643300	-1.52805100	-4.98707200
H	-3.47991000	-1.02954900	-5.38584800
H	-4.86936200	-0.84327400	-4.29813600
H	-5.04389300	-1.72974200	-5.82201000
O	1.43329400	-0.13026300	-1.72985700
N	2.46385800	-0.53375000	-2.40900200
C	2.29204700	-1.20240100	-3.57332500
H	1.25959300	-1.32917600	-3.87599800
C	3.38556800	-1.68869500	-4.27022300
H	3.21092800	-2.23085200	-5.19272700
C	4.66951600	-1.48872000	-3.78205400
H	5.53210900	-1.87734800	-4.31415200
C	4.84397500	-0.76319200	-2.59918100
C	3.71688900	-0.29075700	-1.94399200
H	3.74847900	0.28446700	-1.02570000
C	6.21117500	-0.47971600	-2.04187400
H	6.80781800	0.08097500	-2.76627200
H	6.73731000	-1.41513400	-1.82797500
H	6.15257300	0.09746000	-1.11712300
O	-2.05357000	0.07319000	4.96835800
N	-2.41121600	0.48075000	3.76332600
C	-2.98287700	1.43307300	1.25000000
H	-3.20037000	1.80787600	0.25348400
C	-2.33933400	0.21310400	1.41383300
H	-2.02634300	-0.38137900	0.56290300

C	-2.06908200	-0.25500200	2.68593300
H	-1.56803300	-1.18988400	2.90731000
C	-3.06411300	1.65770500	3.62668900
H	-3.28571200	2.15708000	4.56060200
C	-3.35267800	2.17836000	2.37212200
C	-3.98747000	3.53419300	2.22401300
H	-4.68255300	3.54729800	1.38062500
H	-3.21626900	4.28405700	2.01402500
H	-4.51968700	3.83130800	3.12954300
O	2.99989100	4.73370000	1.86683400
N	3.56991900	3.71862700	2.41099700
C	4.59480800	3.08613700	1.77962300
H	4.87901000	3.53011800	0.83519000
C	5.19504300	1.97631100	2.34444900
H	6.00639000	1.49730400	1.80742100
C	4.75247000	1.48669100	3.56807100
H	5.20492900	0.60402000	4.00903300
C	3.71031600	2.14648700	4.22299400
C	3.15006700	3.26219000	3.61963800
H	2.33115700	3.82665300	4.04815900
C	3.19431200	1.68332300	5.55864800
H	3.12605900	0.59295600	5.58653200
H	2.20129100	2.08903000	5.76322300
H	3.86910800	1.99989300	6.35963400

IV Solution studies

A 10 mM stock solution of the *C*-hexyl-2-methylresorcinarene BrC6 host and 20 mM of the guests were prepared in the respective solvents (acetone-d_6, $CD_3OD/CDCl_3$ 1:1 v/v and CD_3OD/DMSO-d_6 9:1 v/v). For the pure host, aliquot 300 µL of the 10 mM stock solution and diluted with the NMR solvent to final volume 450 µL with a concentration 6.6 mM. For the pure guest, aliquot 150 µL of the 20 mM stock solution and diluted to a final volume 450 µL with a concentration 6.6 mM. For the 1:1 host–guest, aliquot 300 µL of host and 150 µL of guest were mixed to final volume 450 µL to give a concentration 6.6 mM.

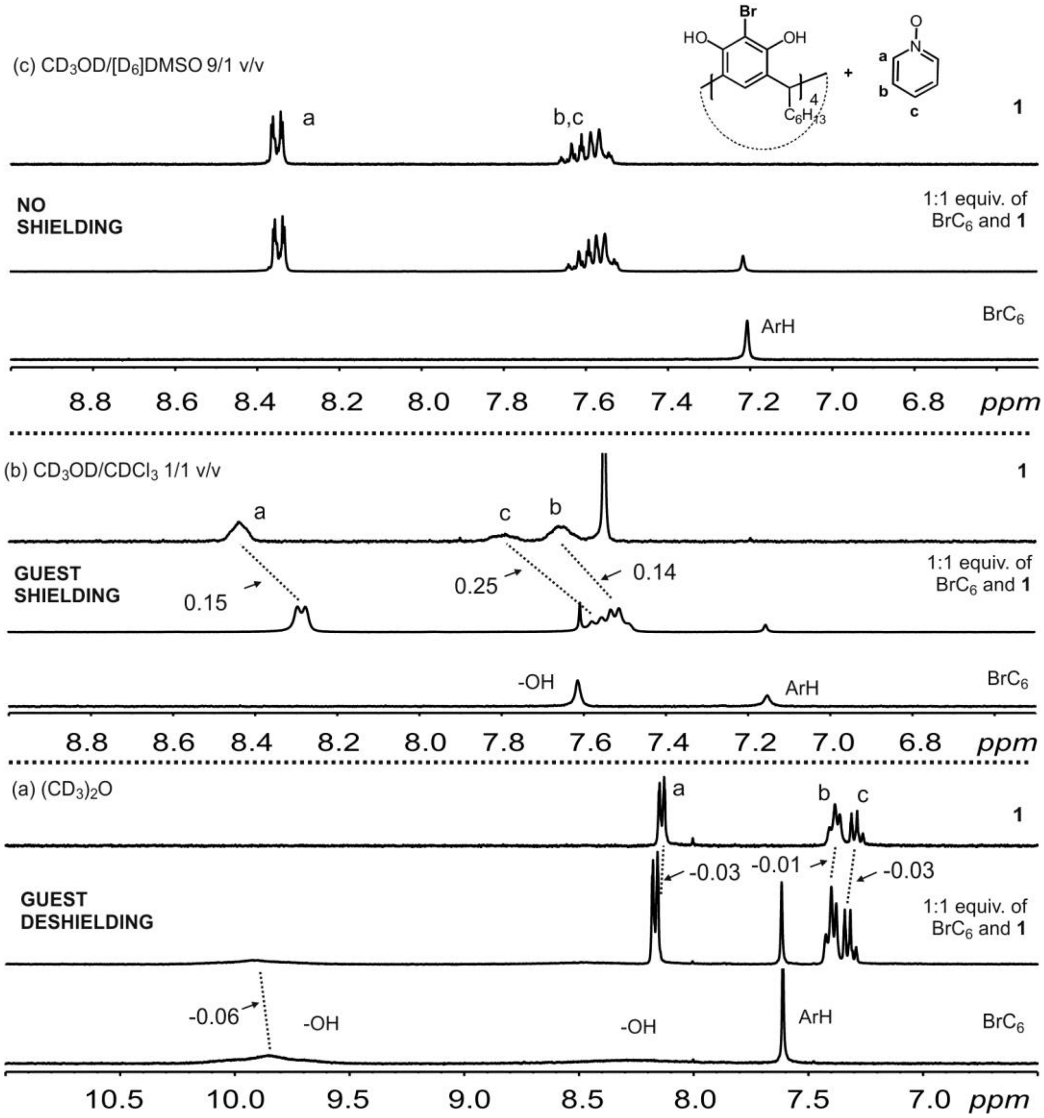

Figure S2: An expansion of the ^{1}H NMR (6.6 mM at 298 K, 500 MHz) of BrC6 complexes with **1**. Spectra are produced from BrC6, **1** and an equimolar mixture of BrC6 and **1** in: (a) $(CD_3)_2O$, (b) $CD_3OD/CDCl_3$ 1:1 v/v, and (c) CD_3OD/DMSO-d_6 9:1 v/v. Dashed lines highlight the observed shift changes of the resonances, labels are in ppm.

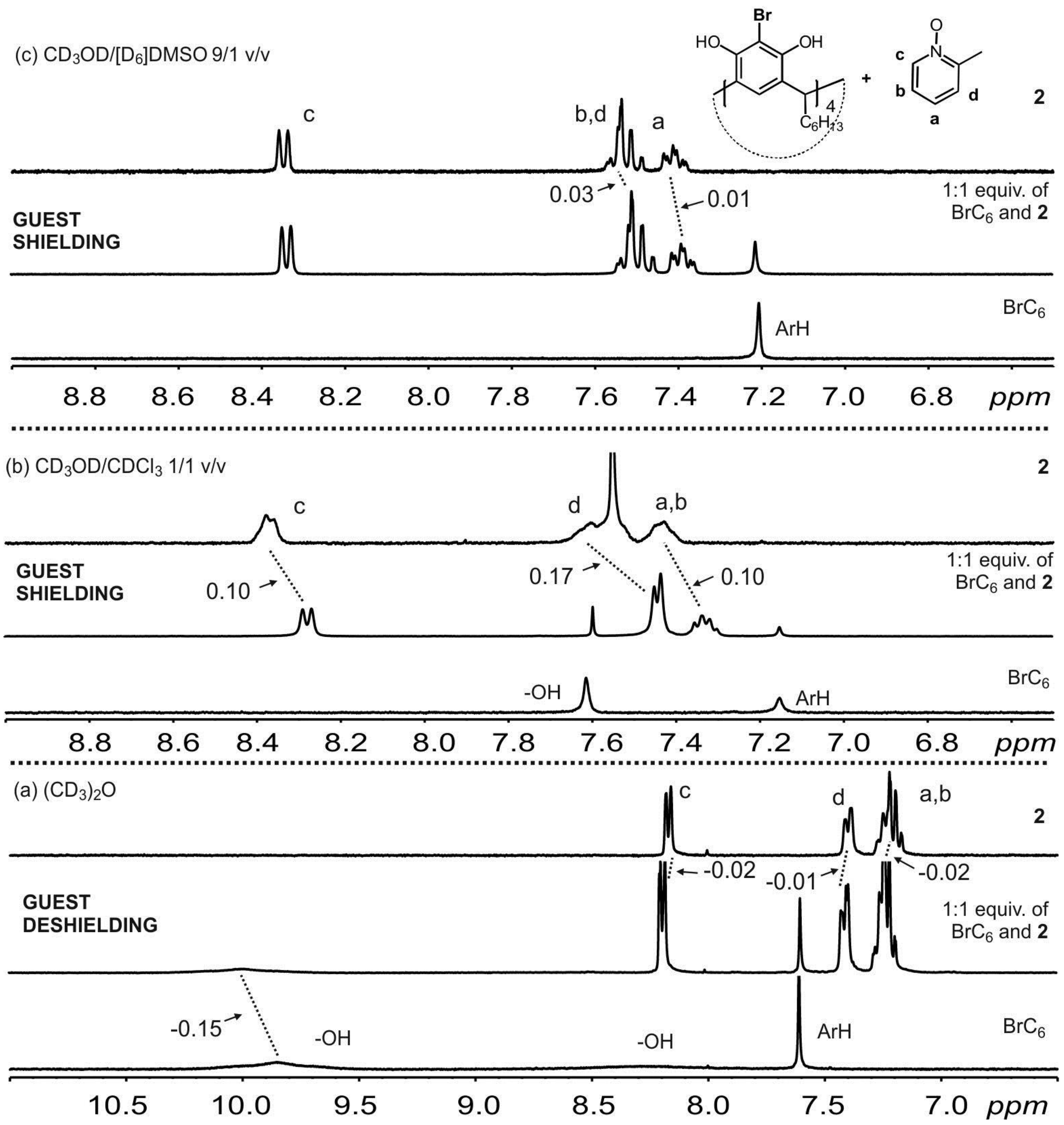

Figure S3: An expansion of the ^{1}H NMR (6.6 mM at 298 K, 500 MHz) of BrC6 complexes with **2**. Spectra are produced from BrC6, **2** and an equimolar mixture of BrC6 and **2** in: (a) $(CD_3)_2O$, (b) $CD_3OD/CDCl_3$ 1:1 v/v, and (c) CD_3OD/DMSO-d_6 9:1 v/v. Dashed lines highlight the observed shift changes of the resonances, labels are in ppm.

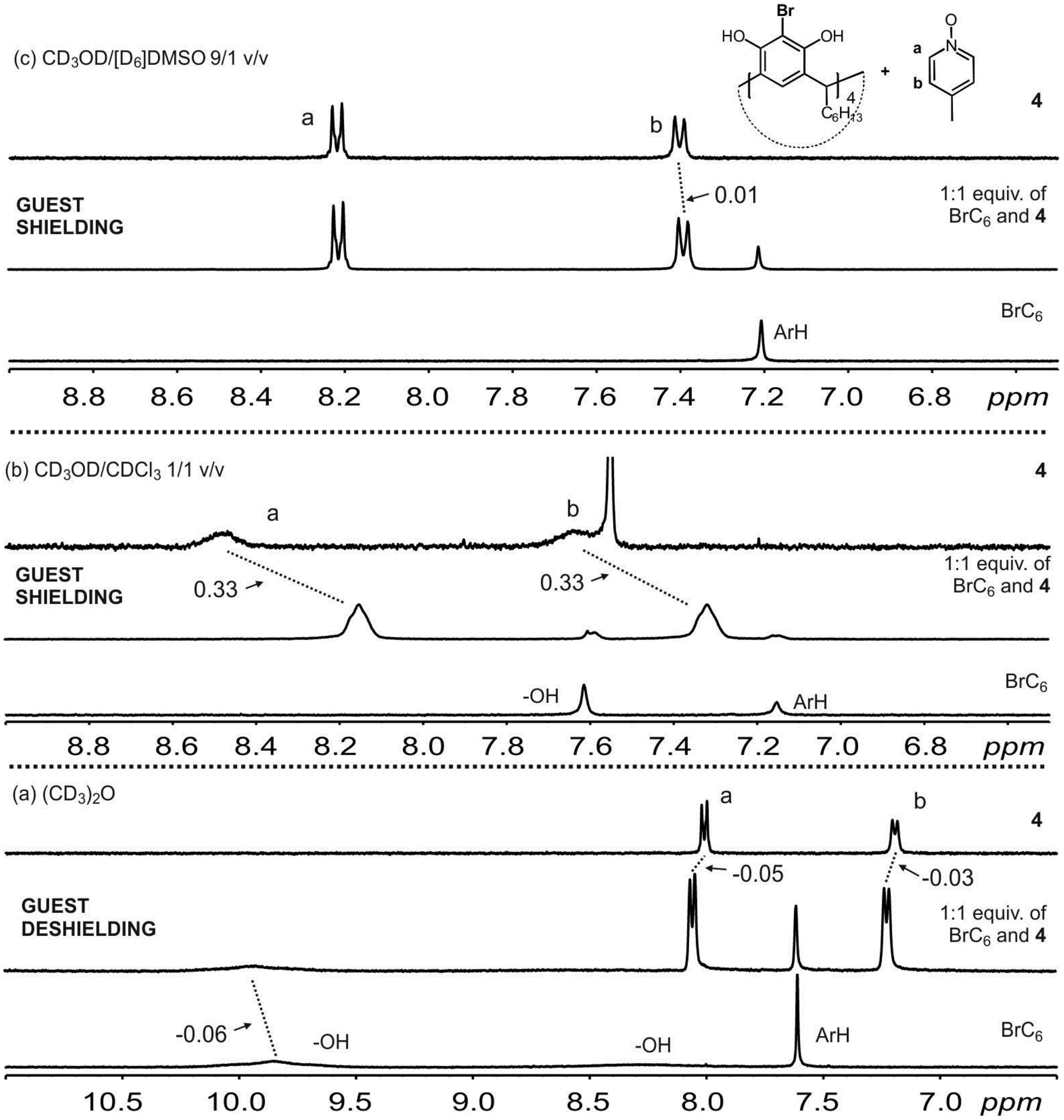

Figure S4: An expansion of the ^{1}H NMR (6.6 mM at 298 K, 500 MHz) of BrC6 complexes with **4**. Spectra are produced from BrC6, **4** and an equimolar mixture of BrC6 and **4** in: (a) $(CD_3)_2O$, (b) $CD_3OD/CDCl_3$ 1:1 v/v, and (c) CD_3OD/DMSO-d_6 9:1 v/v. Dashed lines highlight the observed shift changes of the resonances, labels are in ppm.

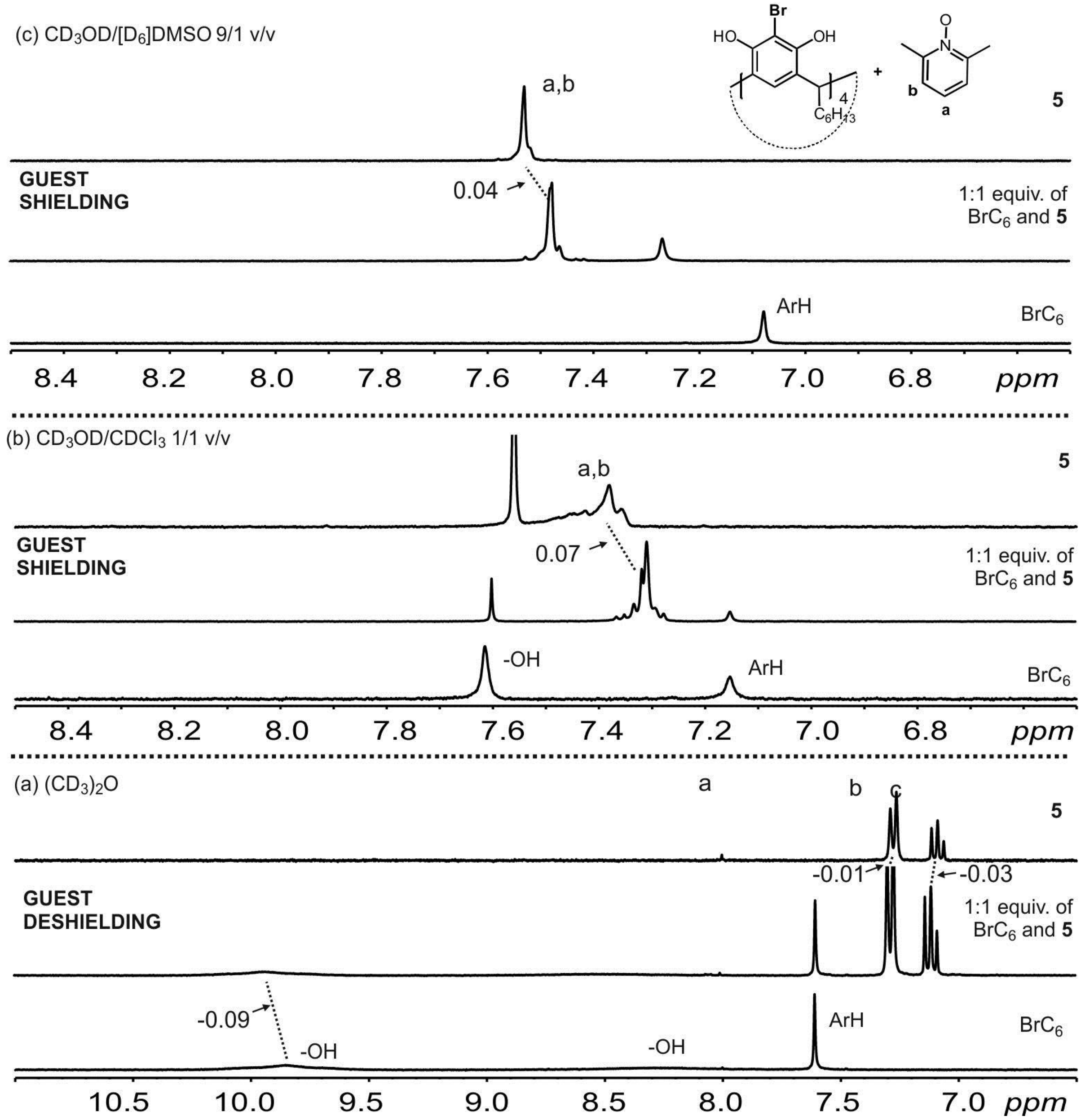

Figure S5: An expansion of the ^{1}H NMR (6.6 mM at 298 K, 500 MHz) of BrC6 complexes with **5**. Spectra are produced from BrC6, **5** and an equimolar mixture of BrC6 and **5** in: (a) $(CD_3)_2O$, (b) $CD_3OD/CDCl_3$ 1:1 v/v, and (c) CD_3OD/DMSO-d_6 9:1 v/v. Dashed lines highlight the observed shift changes of the resonances, labels are in ppm.

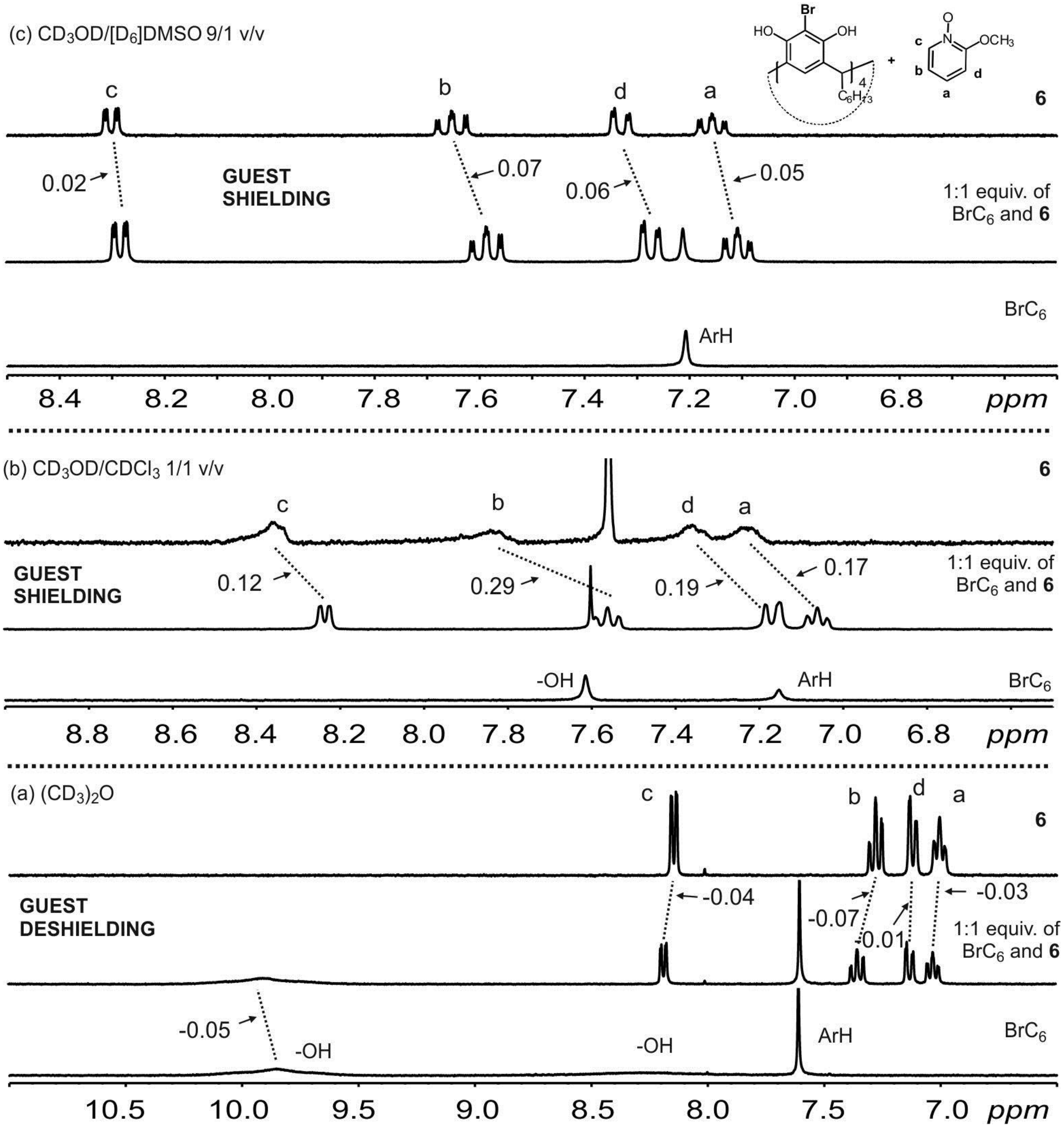

Figure S6: An expansion of the ^{1}H NMR (6.6 mM at 298 K, 500 MHz) of BrC6 complexes with **6**. Spectra are produced from BrC6, **6** and an equimolar mixture of BrC6 and **6** in: (a) $(CD_3)_2O$, (b) $CD_3OD/CDCl_3$ 1:1 v/v, and (c) CD_3OD/DMSO-d_6 9:1 v/v. Dashed lines highlight the observed shift changes of the resonances, labels are in ppm.

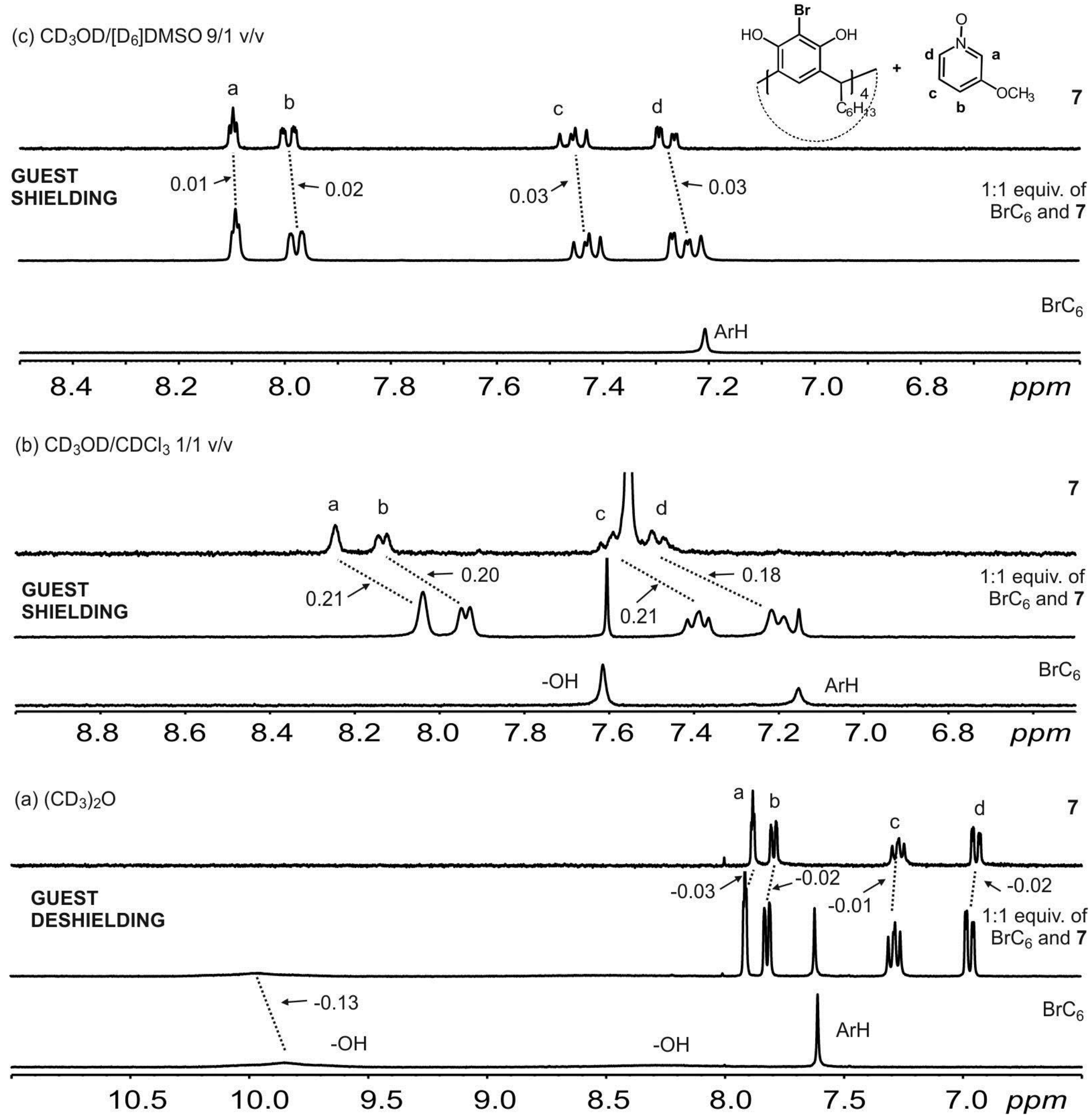

Figure S7: An expansion of the ^{1}H NMR (6.6 mM at 298 K, 500 MHz) of BrC6 complexes with **7**. Spectra are produced from BrC6, **7** and an equimolar mixture of BrC6 and **7** in: (a) $(CD_3)_2O$, (b) $CD_3OD/CDCl_3$ 1:1 v/v, and (c) CD_3OD/DMSO-d_6 9:1 v/v. Dashed lines highlight the observed shift changes of the resonances, labels are in ppm.

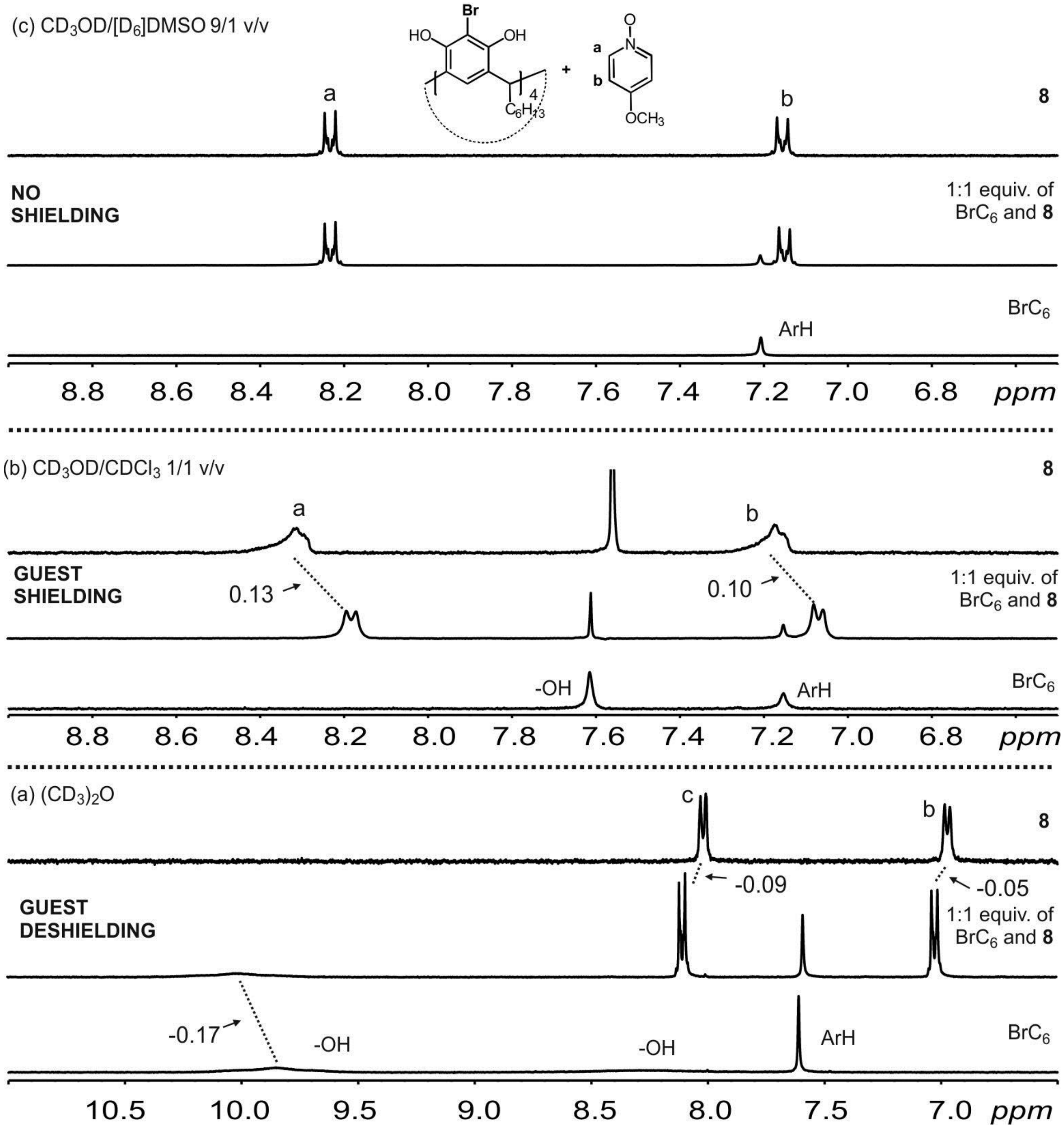

Figure S8: An expansion of the ^{1}H NMR (6.6 mM at 298 K, 500 MHz) of BrC6 complexes with **8**. Spectra are produced from BrC6, **8** and an equimolar mixture of BrC6 and **8** in: (a) $(CD_3)_2O$, (b) $CD_3OD/CDCl_3$ 1:1 v/v, and (c) CD_3OD/DMSO-d_6 9:1 v/v. Dashed lines highlight the observed shift changes of the resonances, labels are in ppm.

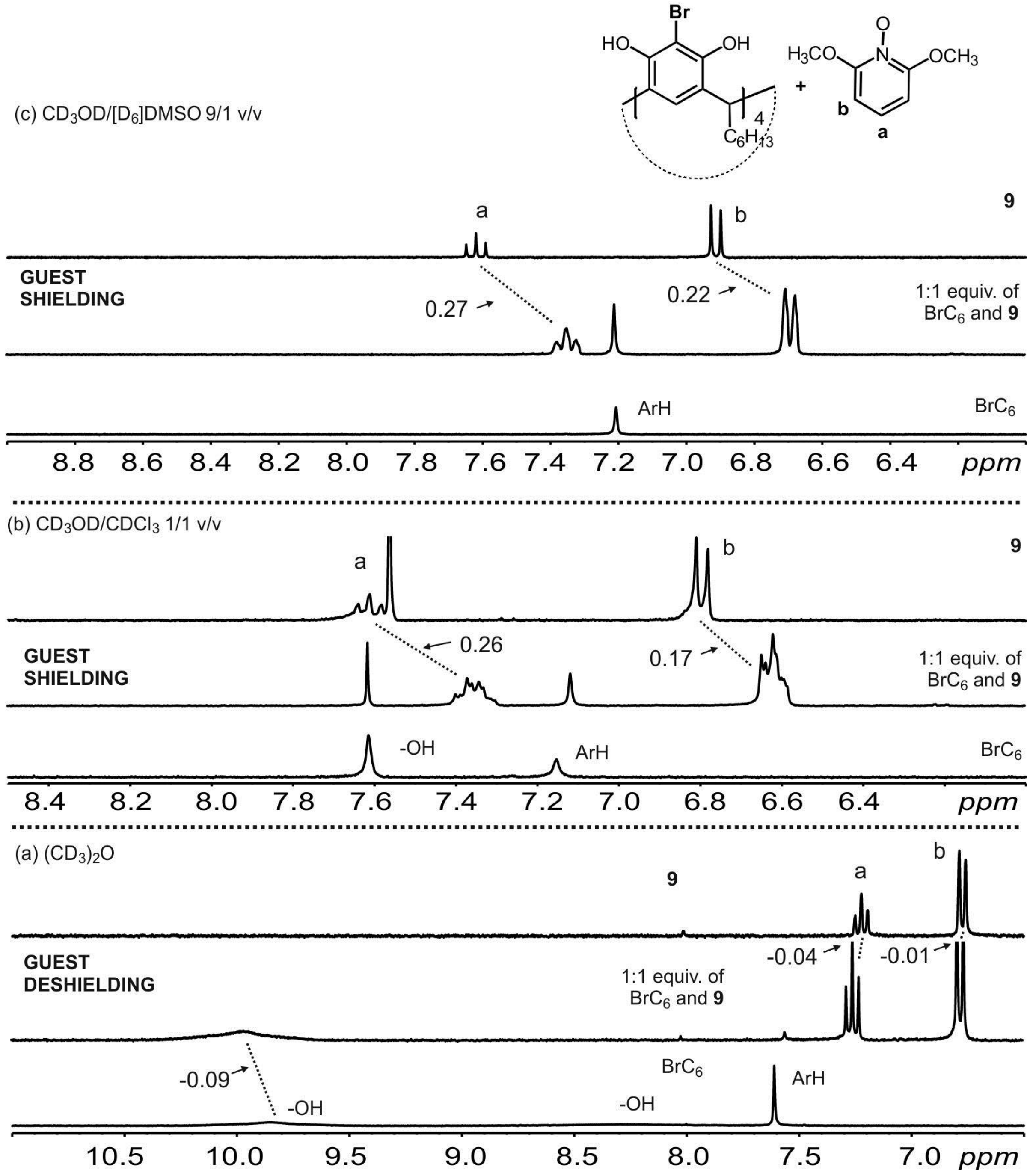

Figure S9: An expansion of the ^{1}H NMR (6.6 mM at 298 K, 500 MHz) of BrC6 complexes with **9**. Spectra are produced from BrC6, **9** and an equimolar mixture of BrC6 and **9** in: (a) $(CD_3)_2O$, (b) $CD_3OD/CDCl_3$ 1:1 v/v, and (c) CD_3OD/DMSO-d_6 9:1 v/v. Dashed lines highlight the observed shift changes of the resonances, labels are in ppm.

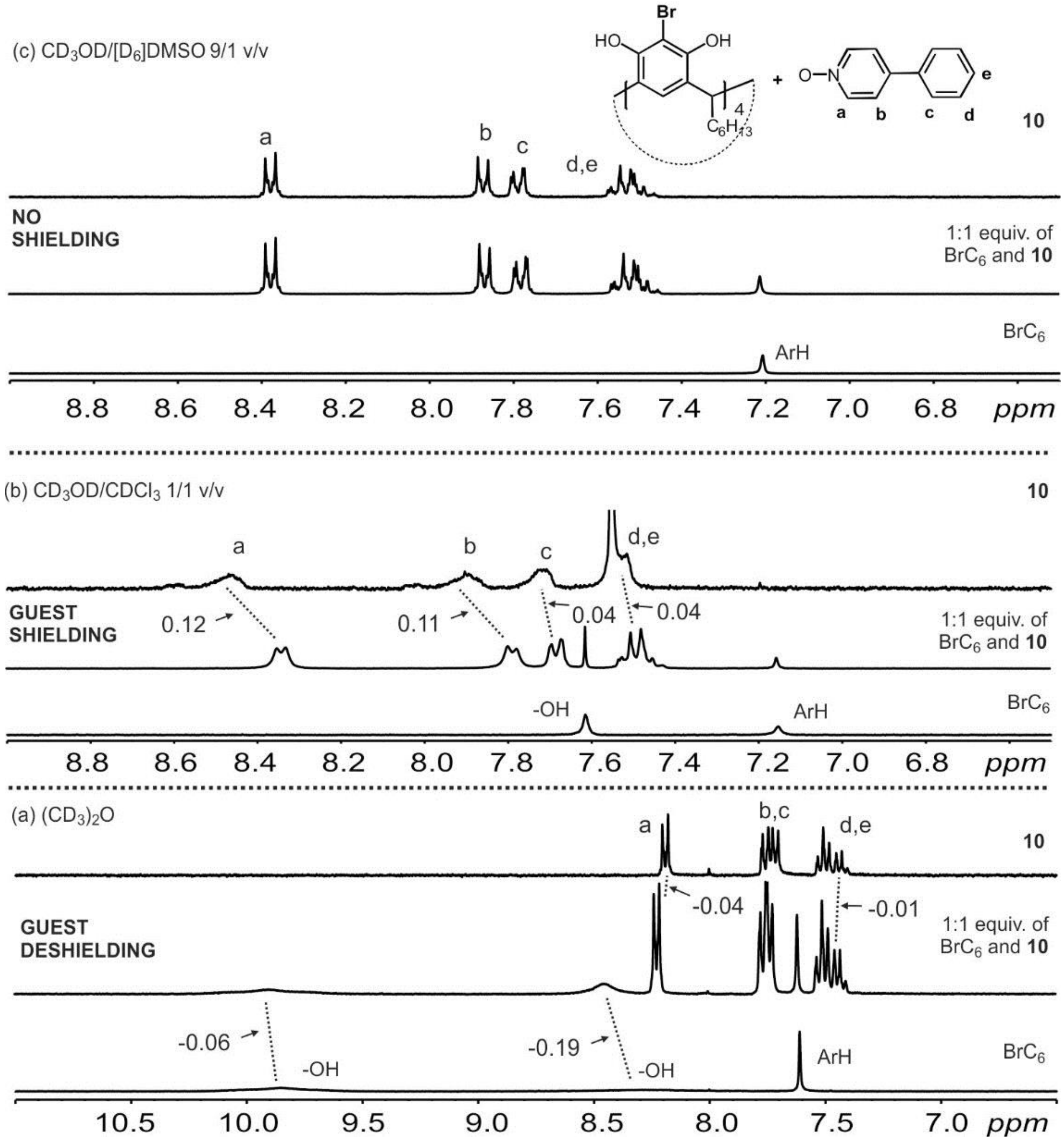

Figure S10: An expansion of the ^{1}H NMR (6.6 mM at 298 K, 500 MHz) of BrC6 complexes with **10**. Spectra are produced from BrC6, **10** and an equimolar mixture of BrC6 and **10** in: (a) $(CD_3)_2O$, (b) $CD_3OD/CDCl_3$ 1:1 v/v, and (c) CD_3OD/DMSO-d_6 9:1 v/v. Dashed lines highlight the observed shift changes of the resonances, labels are in ppm.

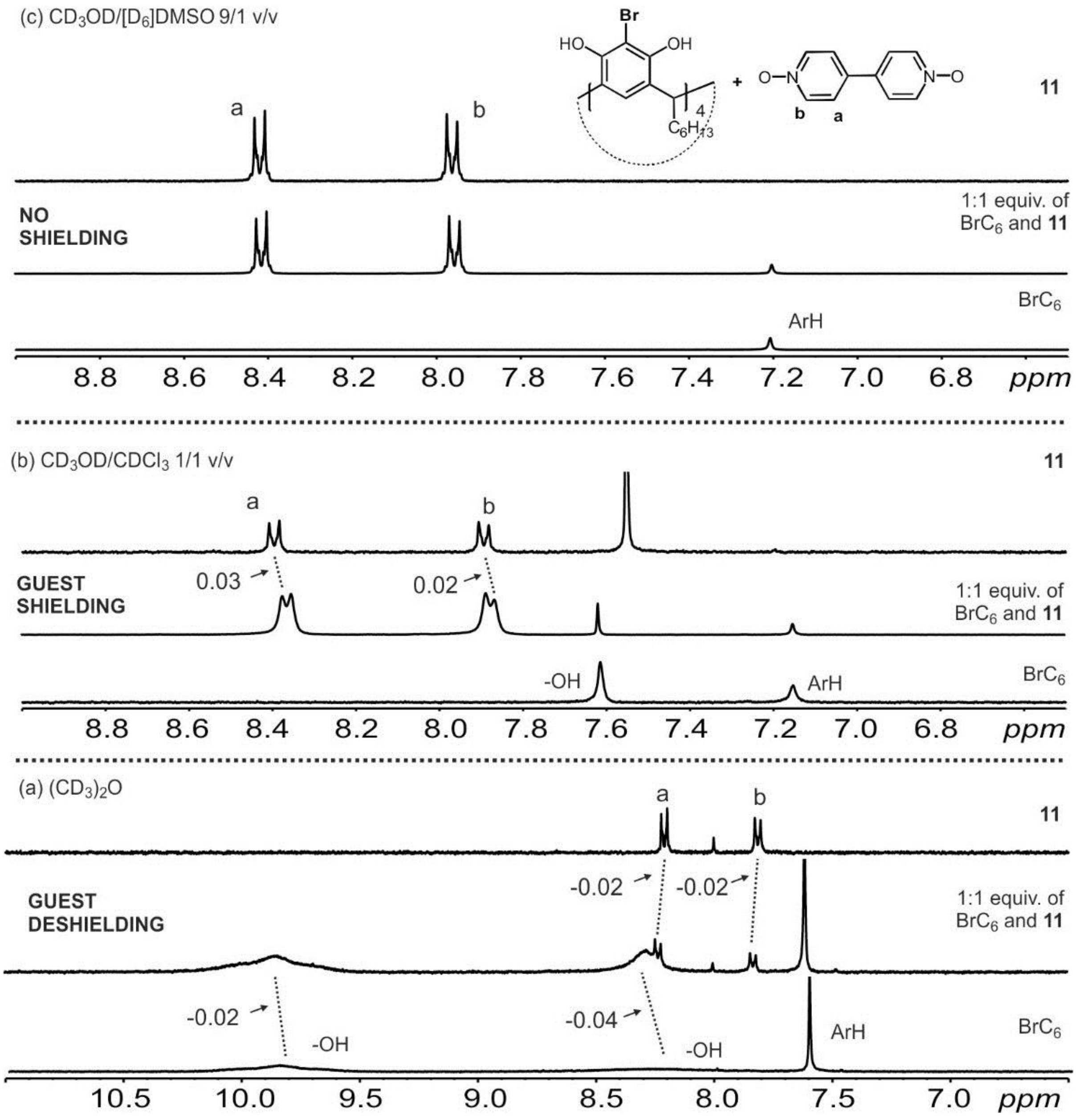

Figure S11: An expansion of the ^{1}H NMR (6.6 mM at 298 K, 500 MHz) of BrC6 complexes with **11**. Spectra are produced from BrC6, **11** and an equimolar mixture of BrC6 and **11** in: (a) $(CD_3)_2O$, (b) $CD_3OD/CDCl_3$ 1:1 v/v, and (c) CD_3OD/DMSO-d_6 9:1 v/v. Dashed lines highlight the observed shift changes of the resonances, labels are in ppm.

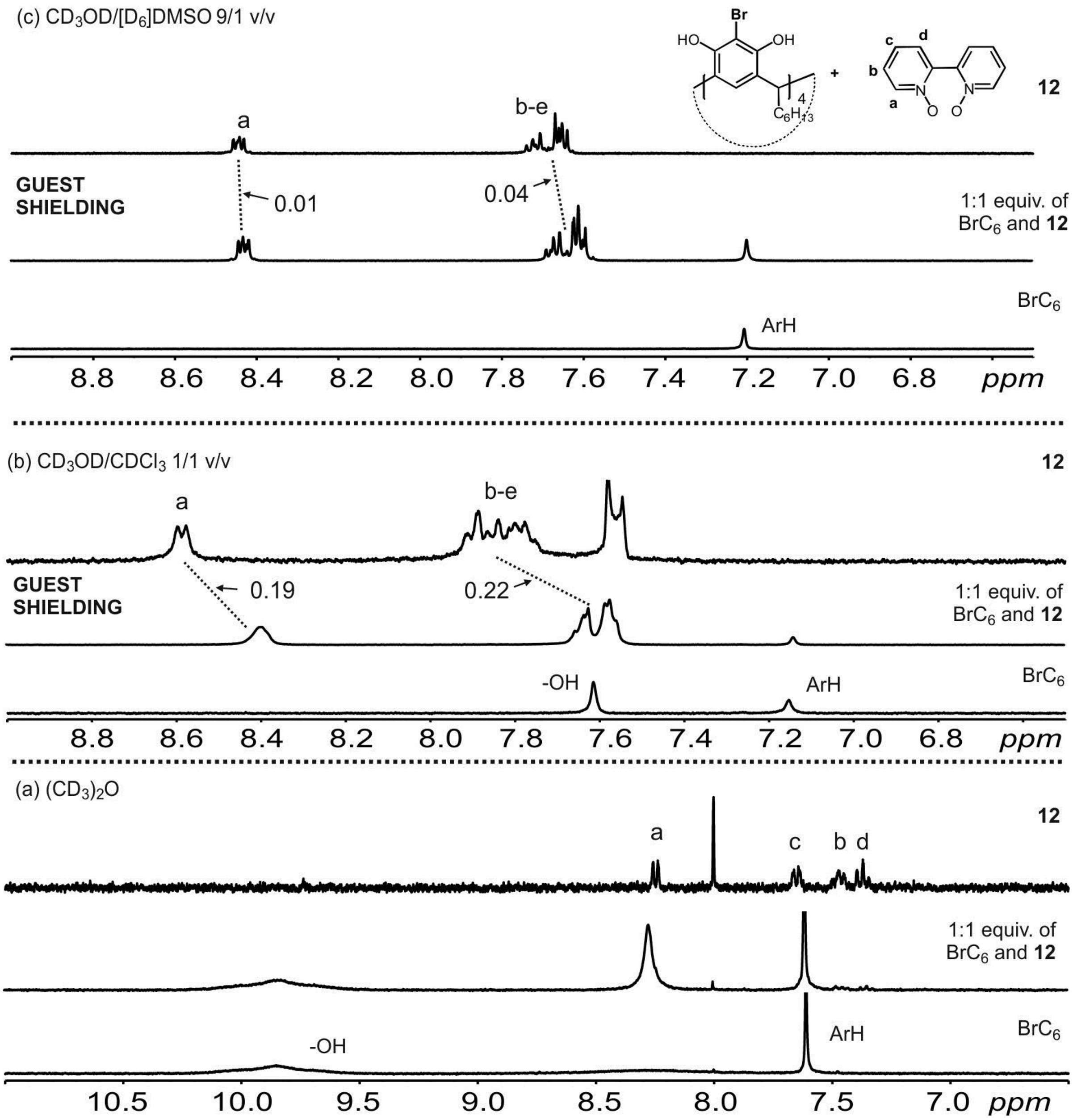

Figure S12: An expansion of the ^{1}H NMR (6.6 mM at 298 K, 500 MHz) of BrC6 complexes with **12**. Spectra are produced from BrC6, **12** and an equimolar mixture of BrC6 and **12** in: (a) $(CD_3)_2O$, (b) $CD_3OD/CDCl_3$ 1:1 v/v, and (c) CD_3OD/DMSO-d_6 9:1 v/v. Dashed lines highlight the observed shift changes of the resonances, labels are in ppm. No analysis of between BrC6 and **12** in acetone-d_6 due to limited solubility of **12** at 6.6 mM.

V References

1. Beyeh N. K, Weimann D. P, Kaufmann L, Schalley C. A, and Rissanen K, *Chem. – A Eur. J.*, **2012**, 18, 5552–5557.
2. (a) Katritzky, A. R.; Lagowski, J. M. *Chemistry of the heterocyclic N-oxides*; Organic chemistry; Academic Press, 1971; (b) Albini, A. *Heterocyclic N-oxides*; Taylor & Francis, 1991.
3. Rigaku Oxford Diffraction 2016, *CrysAlisPro* 38.43.
4. *Bruker AXS BV*, Madison, WI, USA; *1997–2004.*
5. Otwinowski, Z. and Minor, W. *Methods Enzymol.* **1997**, *276*, 307–326.
6. Blessing, R. H. *J. Appl. Cryst.* **1997**, *30*, 421–426.
7. Sheldrick, G.M. *Acta Cryst. A* **2015**, *71*, 3–8
8. Dolomanov, O. V.; Bourhis, L. J.; Gildea, R. J.; Howard, J. A. K.; Puschmann, H. *J. Appl. Cryst.* **2009**, *42*, 339–341.
9. (a) Schrödinger Release 2017-2: MacroModel, Schrödinger, LLC, New York, NY, 2017; (b) Schrödinger Release 2017-2: Maestro, Schrödinger, LLC, New York, NY, 2017.
10. Frisch, M. J.; Trucks, G. W.; Schlegel, H. B.; Scuseria, G. E.; Robb, M. A.; Cheeseman, J. R.; Scalmani, G.; Barone, V.; Mennucci, B.; Petersson, G. A.; Nakatsuji, H.; Caricato, M.; Li, X.; Hratchian, H. P.; Izmaylov, A. F.; Bloino, J.; Zhang, G.; Sonnenberg, J. L.; Hada, M.; Ehara, M.; Toyota, K.; Fukuda, R.; Hasegawa, J.; Ishida, M.; Nakajima, T.; Honda, Y.; Kitao, O.; Nakai, H.; Vreven, T.; Montgomery, J. A.; Peralta, Jr., J. E.; Ogliaro, F.; Bearpark, M.; Heyd, J. J.; Brothers, E.; Kudin, K. N.; Staroverov, V. N.; Kobayashi, R.; Normand, J.; Raghavachari, K.; Rendell, A.; Burant, J. C.; Iyengar, S. S.; Tomasi, J.; Cossi, M.; Rega, N.; Millam, J. M.; Klene, M.; Knox, J.e.; Cross, J. B.; Bakken, V.; Adamo, C.; Jaramillo, J.; Gomperts, R.; Stratmann, R. E.; Yazyev, O.; Austin, A. J.; Cammi, A. R.; Pomelli, C.; Ochterski, J. W.; Martin, R. L.; Morokuma, K.; Zakrzewski, V. G.; Voth, G. A.; Salvador, P.; Dannenberg, J. J.; Dapprich, S.; Daniels, A. D.; Farkas, Ö.; Foresman, J. B.; Ortiz, J. V.; Cioslowski, J.; Fox, D. J. Gaussian 09, Revision D.02; Gaussian, Inc., Wallingford, CT, 2009.
11. Walker, M.; Harvey, A. J. A.; Sen, A.; Dessent, C. E. H. *J. Phys. Chem. A* **2013**, *117*, 12590–12600.
12. Tomasi, J.; Mennucci, B.; Cances, E. *J. Mol. Struct.: THEOCHEM* **1999**, *464*, 211–226.
13. Chai, J.-D.; Head-Gordon, M. *Phys. Chem. Chem. Phys.* **2008**, *10*, 6615–6620.
14. Bader, R. F. W. Atoms in Molecules, A Quantum Theory; Oxford University Press: Oxford, 1990
15. (a) Espinosa, E.; Molins, E.; Lecomte, C. *Chem. Phys. Lett.* **1998**, *285*, 170–173; (b) Yurenko, Y. P.; Zhurakivsky, R. O.; Samijlenko, S. P.; Ghomi, M.; Hovorun, D. M. *Chem. Phys. Lett.* **2007**, *447*, 140–146.
